# Supplementary material for: Protocol for a multicentre randomised controlled trial of STeroid Administration Routes For Idiopathic Sudden sensorineural Hearing loss: The STARFISH trial
Source: PLoS One. 2024 Feb 29;19(2):e0290480. doi: 10.1371/journal.pone.0290480 (PMC10903811; doi:10.1371/journal.pone.0290480)
Supplement: S1 File — (PDF) [file pone.0290480.s002.pdf]

Supporting Information Section 2: Outcome Assessment Procedures

Pure tone audiogram

Pure tone audiometry will be undertaken in clinics by audiologists blind to treatment allocation, in accordance with the British Society of Audiology Recommended Procedure for pure-tone air-conduction and bone conduction threshold audiometry with and without masking (2018). Details can be found at: https://www.thebsa.org.uk/resources/pure-tone-air-bone-conduction-threshold-audiometry-without-masking/.

AB word speech testing

Audiologists blind to treatment allocation will perform speech testing, using AB wordlists on each ear individually. Supra-aural headphones (TDH-39) are routinely used, but insert earphones may be selected at the audiologist's discretion, taking care to select the appropriate routing and masking levels.

The ear with the best audiometric thresholds will be tested first to allow the participant to become familiarised with the task. The initial presentation level (Decibel Sensation Level, above PTA) will be around 30-40 dB above the PTA average across 250, 500 and 1000Hz. Masking of the contralateral ear will be applied in order to prevent the non-test ear being able to hear the speech material presented to the test ear. If the participant has a sensorineural hearing loss the masking noise will be presented 30 dB below the speech signal. The appropriate level of masking noise for sensorineural, conductive and mixed losses is derived from the following equation (Coles and Priede 1975):

Ds + Em + max ABG nt -40

Where:

- Ds is the dial setting for presentation of speech to the test ear
- Em is effective masking calculated by measuring the difference in dial setting for speech presented to normal listeners at a level giving over 95% in quiet and the dial setting of noise presented to the same ear which leads to speech scores less than 10%. This factor has previously been measured and found to be 10 dB using a local biological calibration
- Max ABG nt is the maximum air-bone gap in the non-test ear 250 to 4000 Hz
- '40' comes from minimum interaural attenuation for masking in audiometry using headphones (note for inserts this would be 55 dB).

Each word is scored as a maximum of 3, one point for each correct phoneme. For the purpose of the study, the aim is to establish the Maximum Recognition Score (MRS), also known as PBmax for a phonemically balanced word list. Following completion of the first list, the presentation level should be increased in 10dB steps above the initial presentation level until one of the following:

- 3 points are obtained with scores over 95%
- Roll-over is clearly identified (a decrease in score at a minimum of two levels beyond the MRS)
- The maximum output of the audiometer is reached

- The participant reports discomfort

Training will be provided for audiologists not familiar with AB word testing. This will be recorded on a training log which should be kept in the ISF. This will take the form of written material and a short video on the testing technique. For any audiologists seeking additional help, remote one to one training will be provided via videoconferencing software. The AB word training material is available at: https://entintegrate.co.uk/starfish.

Online hearing tests

Bespoke software has been developed for the trial by the HearX Group (Pretoria, South Africa) to allow regular interval testing to chart the recovery of hearing. These tests are accessed via the trial website, and will allow participants to self-test their hearing online at home. It will be recommended that participants complete the online tests weekly for the 12 week follow up period, however this part of the trial will be optional. Participants without internet access or the required skills, or those who prefer not to test at home will still be able to participate in the trial.

On visiting the website participants will be asked to enter their unique identifier provided at randomisation, and to connect headphones to the computer or device they are using. Headphones will be provided if participants do not have access to their own.

First participants will indicate the severity of their dizziness and the severity of their tinnitus on a 0-10 visual analogue scale (VAS) using a slider. Next participants will complete a digits in noise test that has been widely used in a World Health Organisation hearing screening app. The trial version has been modified to allow the collection of ear-specific data. Data from both ears will be collected, with the contralateral ear providing a measure of test-retest variability. Next participants will complete pure tone audiometry, again collecting ear-specific thresholds.

It will be recommended that participants use the same location and approximate time for the hearing tests, in an attempt to improve the consistency of results and to aid in regular testing. HearX has significant experience with digital remote hearing assessment, however additional validation procedures have been included in the study analysis plan to assess if online thresholds accurately reflect in-hospital hearing test results. Online hearing tests conducted within a few days of the pre-injection pure tone audiogram tests and the 6 and 12 week pure tone and speech tests will be compared to these audiologist-performed in-hospital hearing tests.
