# Supplementary material for: Protocol for a multicentre randomised controlled trial of STeroid Administration Routes For Idiopathic Sudden sensorineural Hearing loss: The STARFISH trial
Source: PLoS One. 2024 Feb 29;19(2):e0290480. doi: 10.1371/journal.pone.0290480 (PMC10903811; doi:10.1371/journal.pone.0290480)
Supplement: S2 File — (PDF) [file pone.0290480.s003.pdf]

Supporting Material Section 3: Adverse event reporting

Adverse Event (AE) Definitions

**Severity Definitions**	Mild<br><br>Moderate<br><br>Severe	Awareness of signs or symptoms that do not interfere with the participant's usual activity or are transient and resolved without treatment and with no sequelae.<br>A sign or symptom, which interferes with the participant's usual activity.<br>Incapacity with inability to do work or perform usual activities.
**Adverse Event**	AE	Any untoward medical occurrence in a participant or clinical trial subject administered a medicinal product and which does not necessarily have a causal relationship with this treatment.<br>Comment:<br>*An AE can therefore be any unfavourable and unintended sign (including abnormal laboratory findings), symptom or disease temporally associated with the use of an investigational medicinal product, whether or not related to the investigational medicinal product.*
**Adverse Reaction**	AR	All untoward and unintended responses to an IMP related to any dose administered.<br>Comment:<br>*An AE judged by either the reporting Investigator or Sponsor as having causal relationship to the IMP qualifies as an AR. The expression reasonable causal relationship means to convey in general that there is evidence or argument to suggest a causal relationship.*
**Serious Adverse Event**	SAE	Any untoward medical occurrence or effect that:<br>● Results in death<br>● Is life-threatening*<br>● Requires hospitalisation or prolongation of existing hospitalisation<br>● Results in persistent or significant disability or incapacity<br>● Is a congenital anomaly/birth defect<br>● Or is otherwise considered medically significant by the Investigator**
**Serious Adverse Reaction**	SAR	An Adverse Reaction which also meets the definition of an SAE
**Unexpected Adverse Reaction**	UAR	An AR, the nature or severity of which is not consistent with the applicable product information (e.g. Investigator Brochure (IB) for an unapproved IMP or (compendium of) Summary of Product Characteristics (SPC) for a licensed product).

		When the outcome of an AR is not consistent with the applicable product information the AR should be considered unexpected.
**Suspected Unexpected Serious Adverse Reaction**	SUSAR	A SAR that is unexpected i.e. the nature, or severity of the event is not consistent with the applicable product information.<br>A SUSAR should meet the definition of an AR, UAR and SAR.

Table S1: Adverse event reporting definitions
* The term life-threatening is defined as diseases or conditions where the likelihood of death is high unless the course of the disease is interrupted.
** Medical events that may not be immediately life-threatening or result in death or hospitalisation but may jeopardise the participant or may require intervention to prevent one of the other outcomes listed in the definitions above.

Adverse Event reporting in STARFISH
The safety profile for this trial population and interventions are well characterised so a strategy of targeted reporting of AEs will not affect the safety of participants.
Only the following AEs will be reported on the 6 week follow up CRF:
- Further sudden deterioration of hearing after intervention
- Affective disorders
- Psychotic reactions
- Stomach ulcer
- Acute ear infection (otitis externa or media)
- New dizziness onset or worsening after intervention
- New tinnitus onset or worsening after intervention
- Persistent ear drum perforation at 6 week review
- Allergic reaction to steroid preparation
- Allergic reaction to local anaesthetic

Only the following AEs will be reported on the 12 week follow up CRF:
- Persistent perforation of the tympanic membrane

The reporting period for AEs will be from commencement of trial treatment until the 12 week follow up visit (see above for further details of timeline requirements of AE reporting). The reporting period for Serious Adverse Events (SAEs) will be from commencement of trial treatment until 6 weeks post treatment commencement.

Serious Adverse Event reporting in STARFISH
All events which meet the definition of serious, must be recorded in the participant notes, including the causality and severity, throughout the participant's time on trial, including follow-up.
For all SAEs, the PI or delegate must do one of the following:
- **Record safety reporting-exempt SAEs** in the medical notes but **not report** them to Birmingham Clinical Trials Unit (BCTU) on an SAE form (1 below).
- **Report SAEs to BCTU in a non-expedited manner**. This can only be done for the pre-defined subset of AEs (2 below).

- **Report SAEs to BCTU in an expedited manner** i.e. within 24 hours of the site research team becoming aware of the event. All SAEs not covered by the above two categories must be reported in this way (3 below).

Note: when an SAE occurs at the same hospital at which the participant is receiving trial intervention or is being followed up for trial purposes, processes must be in place to make the trial team at the hospital aware of any SAEs, regardless of which department first becomes aware of the event, in an expedited manner.

1. Serious Adverse Events not requiring reporting to BCTU

At whatever time they occur during an individual's participation, from consent to end of participant follow up, the following are not considered to be critical to evaluations of the safety of the trial:

a) Pre-planned hospitalisation
b) Hospitalisations lasting less than 24hrs

Such events are "safety reporting exempt".

2. Serious Adverse Events requiring non-expedited reporting to BCTU

Where the safety profile is well established, the causal relationship between the intervention (or the participant's underlying condition) and the SAE, may be known. That is, such events are protocol-defined as "expected".

Such events should still be recorded in the participant's medical notes and reported to BCTU on the SAE form, but it does not require expedited reporting (immediately on the site becoming aware of the event) since the assessment of expectedness for the specified events has been pre-defined. These events are detailed in Table S2.

Table S2: Expected SAEs for the STARFISH trial

**Expected events related to oral steroids**	**Expected events related to intratympanic injection**	**Expected events related to both oral steroids and intratympanic injection**
Blood glucose derangement requiring hospitalisation	Complete sensorineural hearing loss following injection	Allergic reaction requiring hospital treatment
Psychotic or affective disorder requiring hospitalisation		

3. Serious Adverse Events requiring expedited reporting to BCTU

All SAEs not listed in sections 1 and 2 must be reported to BCTU on a trial specific SAE form, within 24 hours of the site research team becoming aware of the event.

SAE Reporting Process

On becoming aware that a participant has experienced an SAE which requires reporting on an SAE form, the PI or delegate should report SAE to their own hospital Trust in accordance with local practice and to BCTU.

To report an SAE to BCTU, the PI or delegate must complete, date and sign an SAE form via the STARFISH trial database. Any other relevant anonymised documents should be submitted to BCTU via the STARFISH trial mailbox (STARFISH@trials.bham.ac.uk). The PI or delegate should also email STARFISH@trials.bham.ac.uk to make BCTU aware that an SAE has been submitted, along with any other relevant anonymised documentation.

Where an SAE Form has been completed by someone other than the PI (or medically qualified delegate) initially, the original SAE form will be required to be countersigned by the PI (or medically qualified delegate) to confirm agreement with the causality and severity assessments.

- Assessment of causality of an SAE

When completing the SAE form, the PI (or, throughout this section, a medically qualified delegate) will be asked to define the nature of the severity and causality of the event.

- Assessment of expectedness of an SAE by a co-CI

The co-CI or delegate will assess all SARs for expectedness with reference to the criteria in Table S3. If the event is unexpected, it will be classified as a Suspected Unexpected Serious Adverse Reaction (SUSAR). The co-CI will review all SAEs submitted and may request further information from the site research team for any given event to assist in this review.

Table S3: Definition of Expectedness

Category	Definition
**Expected**	An adverse event that is consistent with known information about the trial related procedures or that is clearly defined in the reference safety information:<br>- Summary of product characteristics (SmPC) for oral prednisolone<br>- SmPC for dexamethasone
**Unexpected**	An adverse event that is <u>not</u> consistent with known information about the trial related procedures or that is <u>not</u> clearly defined in the reference safety information.

- Provision of follow-up information

Following reporting of an SAE for a participant, the participant should be followed up until resolution or stabilisation of the event. Follow-up information should be provided to BCTU via the trial database. The site should also email the trial mailbox to inform BCTU that follow up information has been submitted.

Reporting SAEs to Third Parties

- Data Monitoring Committee (DMC)

The independent DMC may review any SAEs at their meetings.

- MHRA, REC and RGT

BCTU will report details of all SARs (including SUSARs) to the Medicines and Healthcare Products Regulatory Agency (MHRA), Research Ethics Committee (REC) and University of Birmingham Research Governance Team (RGT) annually from the date of the Clinical Trial Authorisation, in the form of a Development Safety Update Report (DSUR).

In addition, BCTU will report a minimal data set of all individual events categorised as a fatal or life threatening SUSAR to the MHRA, REC and RGT within 7 days of being notified. Follow-up information will be provided within an additional 8 days. All other events categorised as non-life threatening/non-fatal SUSARs will be reported within 15 days of being notified. The MHRA, REC and RGT will be notified immediately if a significant safety issue is identified during the course of the trial. Details of all SUSARs and any other safety issues which arise during the course of the trial will be reported to PIs.

Urgent Safety Measures

The Clinical Trials Regulations make provision for the Sponsor and PIs to take appropriate Urgent Safety Measures to protect a research participant from an immediate hazard to their health and safety. This measure can be taken before seeking approval from MHRA and REC. If any urgent safety measures are taken, BCTU shall immediately and in any event no later than 3 days from the date the measures are taken, give written notice to the MHRA and REC of the measures taken and the reason why they have been taken.

Outcome Assessment Procedures

Pure tone audiogram

Pure tone audiometry will be undertaken in clinics by audiologists blind to treatment allocation, in accordance with the British Society of Audiology Recommended Procedure for pure-tone air-conduction and bone conduction threshold audiometry with and without masking (2018). Details can be found at: https://www.thebsa.org.uk/resources/pure-tone-air-bone-conduction-threshold-audiometry-without-masking/.

AB word speech testing

Audiologists blind to treatment allocation will perform speech testing, using AB wordlists on each ear individually. Supra-aural headphones (TDH-39) are routinely used, but insert earphones may be selected at the audiologist's discretion, taking care to select the appropriate routing and masking levels.

The ear with the best audiometric thresholds will be tested first to allow the participant to become familiarised with the task. The initial presentation level (Decibel Sensation Level, above PTA) will be around 30-40 dB above the PTA average across 250, 500 and 1000Hz. Masking of the contralateral ear will be applied in order to prevent the non-test ear being able to hear the speech material presented to the test ear. If the participant has a sensorineural hearing loss the masking noise will be presented 30 dB below the speech signal. The appropriate level of masking noise for sensorineural, conductive and mixed losses is derived from the following equation (Coles and Priede 1975):

Ds + Em + max ABG nt -40

Where:

- Ds is the dial setting for presentation of speech to the test ear

- Em is effective masking calculated by measuring the difference in dial setting for speech presented to normal listeners at a level giving over 95% in quiet and the dial setting of noise presented to the same ear which leads to speech scores less than 10%. This factor has previously been measured and found to be 10 dB using a local biological calibration
- Max ABG nt is the maximum air-bone gap in the non-test ear 250 to 4000 Hz
- '40' comes from minimum interaural attenuation for masking in audiometry using headphones (note for inserts this would be 55 dB).

Each word is scored as a maximum of 3, one point for each correct phoneme. For the purpose of the study, the aim is to establish the Maximum Recognition Score (MRS), also known as PBmax for a phonemically balanced word list. Following completion of the first list, the presentation level should be increased in 10dB steps above the initial presentation level until one of the following:

- 3 points are obtained with scores over 95%
- Roll-over is clearly identified (a decrease in score at a minimum of two levels beyond the MRS)
- The maximum output of the audiometer is reached
- The participant reports discomfort

Training will be provided for audiologists not familiar with AB word testing. This will be recorded on a training log which should be kept in the ISF. This will take the form of written material and a short video on the testing technique. For any audiologists seeking additional help, remote one to one training will be provided via videoconferencing software. The AB word training material is available at: https://entintegrate.co.uk/starfish.

Online hearing tests

Bespoke software has been developed for the trial by the HearX Group (Pretoria, South Africa) to allow regular interval testing to chart the recovery of hearing. These tests are accessed via the trial website, and will allow participants to self-test their hearing online at home. It will be recommended that participants complete the online tests weekly for the 12 week follow up period, however this part of the trial will be optional. Participants without internet access or the required skills, or those who prefer not to test at home will still be able to participate in the trial.

On visiting the website participants will be asked to enter their unique identifier provided at randomisation, and to connect headphones to the computer or device they are using. Headphones will be provided if participants do not have access to their own.

First participants will indicate the severity of their dizziness and the severity of their tinnitus on a 0-10 visual analogue scale (VAS) using a slider. Next participants will complete a digits in noise test that has been widely used in a World Health Organisation hearing screening app. The trial version has been modified to allow the collection of ear-specific data. Data from both ears will be collected, with the contralateral ear providing a measure of test-retest variability. Next participants will complete pure tone audiometry, again collecting ear-specific thresholds.

It will be recommended that participants use the same location and approximate time for the hearing tests, in an attempt to improve the consistency of results and to aid in regular testing. HearX has significant experience with digital remote hearing assessment, however additional validation procedures have been included in the study analysis plan to assess if online thresholds accurately reflect in-hospital hearing test results. Online hearing tests conducted within a few days of the pre-injection pure tone audiogram tests and the 6 and 12 week pure tone and speech tests will be compared to these audiologist-performed in-hospital hearing tests.
