# Supplementary material for: Protocol for a multicentre randomised controlled trial of STeroid Administration Routes For Idiopathic Sudden sensorineural Hearing loss: The STARFISH trial
Source: PLoS One. 2024 Feb 29;19(2):e0290480. doi: 10.1371/journal.pone.0290480 (PMC10903811; doi:10.1371/journal.pone.0290480)
Supplement: S3 File — (PDF) [file pone.0290480.s004.pdf]

<local headed letterhead>

Participant Information Sheet

STARFISH: **ST**eroid **A**dministration **R**outes **F**or **I**diopathic **S**udden Sensorineural **H**earing Loss

An introduction

The patients who are working with us on this study have stressed how extremely distressing and debilitating sudden unexplained hearing loss has been for them and so we appreciate how anxious you are feeling. To go abruptly from normal hearing to sudden deafness, possibly with dizziness and ringing in your ears is very disturbing. The big question for all our patients is "Will I get my hearing back?". While we cannot promise you that treatment will restore your hearing, we want to reassure you that there are some things which can be done for you and treatment is available.

Whilst the causes of this form of hearing loss are not well understood, we do have steroid treatments that can be effective in restoring your lost hearing. These treatments are used regularly in the NHS and include:

1) Steroid tablets taken by mouth
2) Steroid injections
3) A combination of both the above

Currently the treatment you are offered will vary depending on where and when you are treated. Our challenge is that we don't yet know which is the most effective way to give you the steroids to get the best hearing recovery.

This is where this trial comes in. Our team aims to find out which of the different steroid treatments works best in improving the hearing of patients suffering from your type of hearing loss. The trial will take a group of patients and assign them randomly to one of the three treatment methods commonly used. We will then track their hearing recovery, as well as measuring the impact of the hearing loss on the participants' lives with questionnaires.

If you are interested in taking part in this trial, this sheet and a team member will now explain the exact details and what it will mean for you and then you can decide if you would like to take part. Don't worry, joining the STARFISH study is voluntary and if you decide not to participate you will still receive our best care and the treatment we normally offer at this hospital.

This Information Sheet

This Participant Information Sheet tells you the purpose of the study, what will happen to you if you take part, and detailed information about how the study takes place. A member of our research team will go through this information sheet with you, to help you to decide whether or not you would like to take part and to answer any questions you may have. We understand that this can be a stressful time so please feel free to talk to others about the study and, if you wish to contact the researchers, details can be found on the final page of this information sheet.

Before you decide if you would like to participate, we would like to give you information about why the research is being done, and what it will involve. This is described in **Section 1**.

If you are interested in taking part after reading Section 1, please continue to **Section 2** which describes who is organising this study and how we will use your information collected during the study.

Study Participant Video

The trial team have created a video to provide more information about the STARFISH trial. You may wish to watch this first, before reading this information sheet. The video, and other material is available at: https://entintegrate.co.uk/starfish-for-patients. *Ask a member of the study team if you would like to watch this video and do not have an internet-enabled device (such as a smartphone) with you.*

Section 1

Invitation

We would like to invite you to take part in our research study, as you have been identified by your medical team as having a sudden unexplained loss of hearing due to damage in the inner ear. We realise that this is a difficult time for you as losing hearing in one ear can be very upsetting and have a big impact on your quality of life. Treatment with steroids can sometimes improve this hearing loss. Joining the study is entirely up to you, and your decision will not affect the standard of care you receive in any way. Before you decide, we would like you to understand why the research is being done and what it will involve for you.

Purpose and background to the research

Sudden loss of hearing due to damage to the cochlea (an inner ear structure that senses sound and sends the information to the brain) can occur without an obvious cause, a condition described as idiopathic sudden sensorineural hearing loss (ISSNHL). This usually occurs in one ear, can range from a mild hearing loss to a completely deaf ear and can be difficult to come to terms with.

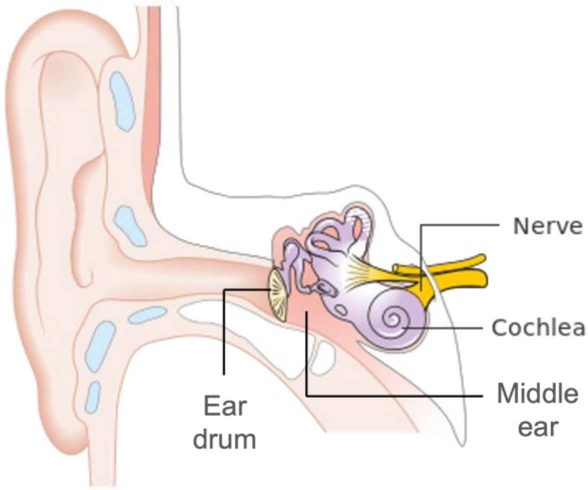

The external, middle and inner ear (cochlea)

The recovery of hearing following ISSNHL may be helped by urgent treatment with steroids, and this is the normal treatment that doctors recommend for this condition. We think that steroids probably work by reducing inflammation in the inner ear.

We are inviting you to volunteer to be chosen at random to receive steroids in one of the three forms which are commonly used in the UK as the usual treatments for sudden hearing loss:

1) Tablets taken by mouth.
2) Injections through the ear drum (with local anaesthetic to numb the area) and into the middle ear space (see diagram above). From here, steroids are taken up into the inner ear.
3) Both tablets by mouth and injections through the ear drum.

At the moment we do not know how best to give steroids for ISSNHL and this study aims to find out which route of steroid treatment works best in improving hearing for patients with ISSNHL. This is a large study that will take place across the UK at around 75 hospitals and over several years. It will eventually involve around 525 patients like you.

The study has two main aims:

1) *To understand which steroid treatment leads to the best recovery of hearing, measured by hearing test results and the impact of the hearing loss on day-to-day life.* The study will also look at other symptoms of ISSNHL, such as ringing sounds in the ears (tinnitus) and dizziness.
2) *To improve the early detection and treatment of ISSNHL.* Many patients currently experience delay in diagnosis and treatment, and this study has been designed to improve the care pathway for patients with a new ISSNHL, with changes to patient care that we hope will continue following the end of the trial.

Why have I been chosen?

We are inviting you to take part in this study because you have had a sudden drop in hearing within the past four weeks where the cause is not known, which has been confirmed by a hearing test. The

medical team looking after you are working with the STARFISH research team, and believe you are able to receive steroid treatment to try to improve your hearing.

What would taking part involve?

When designing this study, we have worked with a group of patients and members of the public to ensure that taking part in the study will not take up too much of your time. A video has been developed together with patients, to help to explain the different parts of this study. It can be found at: https://entintegrate.co.uk/starfish-for-patients.

If you decide to take part having read this information sheet, we will ask you to sign a consent form, and give you a copy of the signed consent form to keep.

Before Treatment:

A member of the research team will ask some questions about your hearing and any other medical problems relevant to this study. Section 2 of this Information Sheet describes the information that we will be collecting.

Next, we will ask you to complete five short questionnaires to record your current hearing ability, any dizziness or tinnitus (ringing in the ear), and the effects they may be having on your life. This should take no longer than 15-20 minutes.

Finally, you will undergo a hearing test to measure your ability to hear speech. This is different from the test that you have already had to measure how well you can hear beeps, and will take around 10 minutes. The questionnaires and speech hearing tests are not part of normal care for patients with ISSNHL, but they will give us valuable information on how the hearing loss is affecting you. Patients tell us that the recovery of any loss of clarity in hearing speech is important and so we also want to track this in the study.

Steroid Treatment:

The researcher will enter your information into a secure online database, and a computer will then pick one of three different steroid treatments for you at random:

- Steroid tablets taken daily for 7 days; or
- A steroid injection through the ear drum, once weekly for 3 weeks; or
- Both tablets and injections as described above.

The researcher will let you know which route of steroids treatment you will receive. Neither you nor the researcher or clinical team can choose the route of steroid treatment that you are assigned to. This process, known as a randomised trial, ensures there is an equal chance of receiving any of the treatments, and is the best way to ensure the study is a fair comparison between the different treatment types.

Given the urgency of the treatment, it is very likely that you will start your tablets and/or injection very soon after you are first seen.

- Steroid tablets are provided by the hospital pharmacy and can then be taken at home daily, once every day for 7 days.
- Any injections will be performed by the team treating you at the hospital. A local anaesthetic cream or spray is given to make your ear drum numb so that the injection is not usually painful

- this takes between 5 and 30 minutes to work. Using a very thin needle and a microscope, the steroid is then injected through your ear drum and into your middle ear in a few seconds. You will then remain lying down for 30 minutes after the injection so that the steroid is taken up into your inner ear.

- If you have an injection, you will be seen one week after the first injection for a hearing test. If your hearing has not completely recovered, you will be offered a second injection on the same day. You will be seen a week later for another hearing test, and again if your hearing has not completely recovered, you will be offered a third injection on the same day.

Monitoring your progress after treatment:

For participants who have internet access using a computer, smartphone or tablet, the researcher will ask you whether you are willing to test your hearing at home once a week. The STARFISH trial website allows you to test your hearing for tones and speech using headphones supplied free by the STARFISH team, or your own headphones if you prefer. If you agree to this part of the study, the researcher can show you how to use the website to provide your test results. The at-home hearing test is provided by a company, HearX. Section 2 details what data we will share and how we will protect your confidentiality if you take part in this optional part of the study. You can opt out of home hearing testing without affecting your participation in the rest of the study.

All participants will be seen by their research team 6 and 12 weeks after the first treatment. At both of these visits your ears will be examined. You will be asked to repeat the hearing test performed by an audiologist that you had before entering the study, the test measuring speech that you had after entering the study, and complete the five questionnaires you did when joining the study. At your final visit we will also ask you about any side effects that you may have experienced from the treatment and any use of healthcare services for your hearing. We expect the extra questionnaires and questions to take no more than 20 minutes.

The visit at 12 weeks will be the end of your participation in the trial at the hospital.

Should you have any questions, concerns or experience any side effects during your time in the study you will be able to contact a member of the research team.

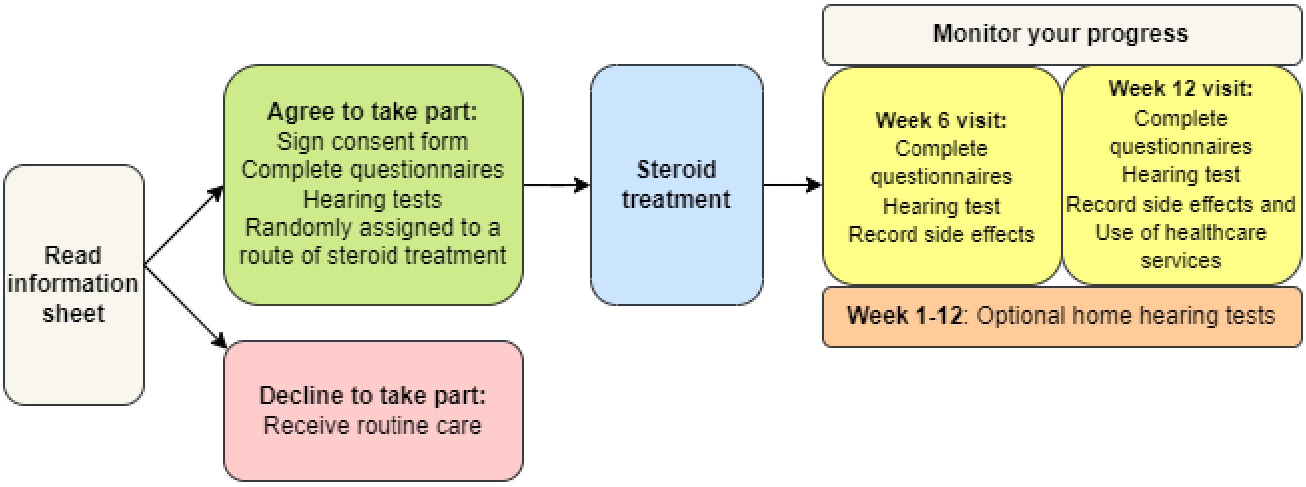

What are the possible benefits of taking part?

Each of the three steroid treatment options are commonly used for ISSNHL. This study will help to find out the best route to give steroids when treating patients with ISSNHL in the future. Whilst there may be no immediate benefits to you, the aim is to improve the future care for people with ISSNHL.

What are the possible disadvantages and risks of taking part?

Many patients participating in this study will not require additional visits to the hospital above those normally required for the treatment of ISSNHL, although this varies between different hospitals. Visits for study participants will be longer than those not in the trial as they will undergo an additional hearing test and complete questionnaires.

Both the tablet and injection forms of steroids used in this study are commonly used in the UK as the usual treatments for ISSNHL and so their safety is well established. Most patients will not experience side effects.

Severe side effects from *tablet* steroid treatment are very rare. Mild side effects are more common but go away once the 7 day treatment is stopped.

Less than half of people:
- Altered sleep (difficulty getting to sleep)
- Mild changes to mood

Less than 1 in 10 people:
- Temporary dizziness
- Indigestion

Very rare, less than 1 in 10,000 people:
- Allergic reaction
- Severe change in mood
- Blood clots
- Bone fracture due to weakness or joint damage
- Spread of infection (sepsis)
- Bleeding from existing stomach ulcers

Severe side effects from the steroid *injection* are also very rare, and most side effects are mild and short-term.

Less than half of people:
- Mild ear discomfort

Less than 1 in 4 people:
- Temporary dizziness for a few minutes

Less than 1 in 20 people:
- Ear infection

Less than 1 in 100 people:
- Persistent hole in the ear drum

Very rare, less than 1 in 1000 people:
- Allergic reaction
- Further hearing loss in the injected ear

What if I become unwell?

The research team will monitor you and your health. If you have any concerns during the study, please contact the research team. If you experience mild side effects such as altered sleep, changes

to mood, dizziness or indigestion then, as long as you feel well, you can continue to take the steroid tablets and complete the course. The course of steroid tablets given in the STARFISH trial can be stopped early if you feel very unwell or experience more severe side effects. For advice please contact the trial team at your local hospital or your GP in the event of problems.

If you experience the very rare side effect of an allergic reaction (which may include a swollen face, tongue or body, and may cause shortness of breath or in very rare cases collapse)**, stop taking the steroid tablets and contact 111 or attend your local Emergency Department.**

We will carefully monitor the side effects that our participants report during the study and an independent safety committee will oversee the study.

What if I have diabetes?

You should discuss taking steroid tablets with a member of the trial team, your GP or practice nurse. Steroid tablets are likely to increase blood glucose level in patients with diabetes and it is important that this is monitored.

What if I am pregnant?

Tell your clinician if you are pregnant, or you could be pregnant. They will discuss any potential risks and you can decide if you would like to participate in the study.

What if something goes wrong?

We do not envisage any problems as a result of your participation in the study. If you have a concern about any aspect of this study, you should ask to speak to a member of the research team who will do their best to answer your questions. Their contact details can be found at the end of this information sheet.

If you remain unhappy and wish to complain formally, the normal National Health Service complaints mechanisms will be available to you. Copies of these guidelines are available on request. If you wish to complain about the way you have been treated during this study, please contact [enter local complaints service] at your local hospital. Contact details can be found on the end of this Information Sheet.

In the event that something does go wrong and you are harmed during the research and this is due to someone's negligence, then you may have grounds for a legal action for compensation against [enter site name], but you may have to pay your legal costs. The normal National Health Service complaints mechanisms will still be available to you (if appropriate).

University of Birmingham, as the study Sponsor, is liable for its employees' actions (undertaken as part of their job) and has insurance against the risk of claims relating to research studies that their staff design and undertake. This study is covered by University of Birmingham's Clinical Trials Insurance.

Involvement of General Practitioner / other healthcare practitioner

With your permission, your GP will be informed of your participation in the study so they are fully aware of your treatment.

What if I do not want to take part?

Participation in this study is entirely voluntary and the standard of care you receive will not be affected if you decide not to take part at any stage. Some aspects of the study are optional, such as the online home hearing tests.

You can stop being part of the study at any time, without giving a reason, but we will keep any information about you that we already gained. Data collected until withdrawal will be used as part of the study outcome.

If you choose to stop any aspect of participation, please speak to a member of the research team. Their contact details can be found at the end of this information sheet.

Section 2

Who is organising and funding the research?

This study is funded by the government through the National Institute for Health Research (NIHR) Health Technology Assessment (HTA) programme (ref: NIHR131528).

The study is sponsored by the University of Birmingham (UoB), which has certain legal and ethical responsibilities for the study (Sponsor study ref: RG_21-145). The study is being coordinated by Birmingham Clinical Trials Unit (BCTU), which is part of UoB. We will collect and process your personal and health data to conduct this study, and UoB is the data controller for this data.

The medical team at your hospital are not being paid for their role in this study.

How have patients and the public been involved in this study?

Both patients with ISSNHL and members of the public helped to develop this study, in particular reducing the number of required hospital visits. Patient representatives are included in the team running the study, and a patient advisory group is regularly consulted. This information sheet and accompanying video have been produced by researchers and patients working in partnership, and a further information booklet and video will be produced with patients to explain the results.

Please note however that only healthcare staff and researchers, and not any patients or members of the public, will have access to your personal records.

Who has reviewed the study?

All research in the NHS is looked at by an independent group of people, called a Research Ethics Committee, to protect your interests. This study has been reviewed and given favourable opinion by Harrow Research Ethics Committee.

Will my taking part in this study be kept confidential?

All information about you and your health will be kept confidential. The only people allowed to look at your information will be the doctors who are running the study, authorised staff at University of Birmingham and your hospital, and the regulatory authorities who check that the study is being carried out correctly. A privacy notice is on the study website: www.birmingham.ac.uk/starfish

If you take part in the study, the following personal information will be transferred and stored at University of Birmingham for use in this study: date of birth, sex, ethnicity, and data about your health and hearing. If you agree, we would also like to use the data we collect to support other related research in the future. This could include sharing the data anonymously with other researchers.

To look at the long-term effects of the treatment, we would like to collect information relating to your hearing for up to 5 years after the trial has ended. The research team may contact you to ask you questions, review your medical notes or look at NHS databases such as NHS digital. If you agree, we will collect your NHS number for this purpose. Allowing this data collection is optional, and opting out of this will not affect your participation in the trial.

If you provide your contact details (name, postal address, e-mail address, mobile phone number), we will use these to send you the final results at the end of the study or to contact you about your participation in the study. If you agree, we would also like to be able to contact you for up to 5 years after the end of the study regarding further data collection, but this is optional.

If you take part in the optional at-home hearing tests, data about your hearing will be held by the company that provides the tests, HearX. To avoid sharing your name, you will have a unique identification number which will allow us to join the data held by HearX with the rest of your study data held at University of Birmingham. If you provide a mobile phone number, we will share this with a company called TextLocal so they can send reminder texts to prompt you to do the at-home hearing tests.

We are also planning to work with HearX to help them develop a new at-home hearing test similar to the Pure Tone Audiogram test that you will do in clinic. If you agree to this optional part of the study, we may send some of your study data to HearX for this purpose. To help protect your confidentiality we will use your unique identification number and not your name. This is a commercial element to this study. You will not benefit financially in any way if commercialisation of any research findings are successful.

Will my travel expenses be reimbursed?

The follow up visits in this study are routine clinic visits that you would be likely to need even if you were not taking part in the study. Unfortunately therefore there is no travel or parking reimbursement available for our participants beyond that normally offered by the hospital.

What happens if new information becomes available?

Sometimes we get new information about the treatment being studied. If this happens, a member of the research team will tell you and discuss whether you should continue in the study.

If your research doctor is happy for you to continue in the study, you will have the option to decide whether you wish to continue. You may be asked to re-sign a consent form if you decide to continue taking part in the study. If you decide not to carry on, a member of the research team will make arrangements for your standard clinical care to continue.

If, however, your research doctor considers that you should withdraw from the study, s/he will explain the reasons and arrange for your standard clinical care to continue.

What happens when the research study stops?

Following your final follow up appointment at 12 weeks after your treatment, a member of the research team will arrange for your standard clinical care to continue.

What will happen to the results of the research study?

The findings of this study will be made public. We would like to publish our results in medical journals, to help other doctors and medical staff to learn from the findings and for patients to benefit. If we are successful with this, the results will be published in an anonymous manner so you cannot be identified.

We also plan to inform all participants of the findings, highlighting where the results are expected to make a clinical difference. This process will include both written material and a video.

How will we use information about you?

We will need to use information from you and your medical records for this research project. This information will include your:

- NHS number
- Name
- Contact details
- Date of birth
- Sex
- Ethnicity

People will use this information to do the research or to check your records to make sure that the research is being done properly.

People who do not need to know who you are will not be able to see your name or contact details.

Your data will have a code number instead.

We will keep all information about you safe and secure.

If you take part in the optional at-home hearing tests, some of your information will be sent to South Africa, where the company that provides the online hearing test are based. They must follow our rules about keeping your information safe. They will not receive your name or contact details.
Once we have finished the study, we will keep some of the data so we can check the results. We will write our reports in a way that no-one can work out that you took part in the study.

What are your choices about how your information is used?

You can stop being part of the study at any time, without giving a reason, but we will keep information about you that we already have.

If you choose to stop taking part in the study, we would like to continue collecting information about your health from your hospital. If you do not want this to happen, tell us and we will stop.

We need to manage your records in specific ways for the research to be reliable. This means that we won't be able to let you see or change the data we hold about you.

If you agree to take part in this study, you will have the option to take part in future research using your data saved from this study.

Where can you find out more about how your information is used?

You can find out more about how we use your information:

- at www.hra.nhs.uk/information-about-patients/
- our leaflet available from www.hra.nhs.uk/patientdataandresearch
- by asking one of the research team
- by contacting the trials team at Birmingham Clinical Trials Unit using the contact details at the end of this information sheet
- at https://www.birmingham.ac.uk/research/bctu/trials/renal/starfish/privacy.aspx

How will my personal data be kept secure?

The University of Birmingham takes great care to ensure that personal data is handled, stored and disposed of confidentially and securely. Our staff receive regular data protection training, and the University has put in place organisational and technical measures so that personal data is processed in accordance with the data protection principles set out in data protection law.

In relation to this project, any physical paperwork containing identifiable data will be kept in an access-controlled and secured room inside a locked filing cabinet. Electronic data will be kept on secure, encrypted IT servers within the University of Birmingham.

If you take part in some optional parts of the study (e.g. the at-home hearing tests) we will also share data with other organisations. We will have legal agreements with any organisation we share your data with and they will also be required to protect your confidentiality and handle your data with care.

How long will my personal data be kept?

Your data will be retained for at least 25 years after the study finishes. If you withdraw from the study, we will keep the information we have already obtained but, to safeguard your rights, we will use the minimum personally-identifiable information possible.

Will I be able to see the results of the study?

If you choose to be contacted, we will send a summary of the study findings to you once the study has finished.

Do you have any further questions?

Thank you for taking the time to read this information sheet and for considering taking part in this study. Should you require further information, would like to speak to someone about the study, or proceed to join the study, please contact:

< Contact Name > <Job Title>

<Telephone and/or E-mail>

< PI Name > <Job Title>

<Telephone and/or E-mail>

For independent advice or support, you can contact the [enter local complaints service]

Tel.: *< PALS Telephone >*   Email: *< PALS E-mail Adress >*

Or you can contact the Chair of the STARFISH trial's independent Steering Committee:

Email: starfish.trial.advice@entintegrate.co.uk

Contact details of STARFISH Trial Office at the Birmingham Clinical Trials Unit:

Email: STARFISH@trials.bham.ac.uk   Website: www.birmingham.ac.uk/starfish
