# Supplementary material for: Protocol for a multicentre randomised controlled trial of STeroid Administration Routes For Idiopathic Sudden sensorineural Hearing loss: The STARFISH trial
Source: PLoS One. 2024 Feb 29;19(2):e0290480. doi: 10.1371/journal.pone.0290480 (PMC10903811; doi:10.1371/journal.pone.0290480)
Supplement: S4 File — (PDF) [file pone.0290480.s005.pdf]

<insert local letterhead details>

INFORMED CONSENT FORM

STARFISH: STeroid Administration Routes For Idiopathic Sudden sensorineural Hearing loss

Patient trial no.: ☐☐☐☐ **Local Principal Investigator:**…………………………………………………………………

EudraCT no.: 2022-000085-17 IRAS no.: 1004878

		Please initial inside each box
1	I confirm that I read and understood the Participant Information Sheet, dated ___ ___ /___ ___ ___ / ___ ___ ___ ___ version number ___ **.** ___ for the STARFISH trial. I had the opportunity to consider the information and ask questions, and have had these answered satisfactorily.	☐
2	I understand that my participation is voluntary and that I am free to withdraw from the trial at any time, without giving any reason, and without my medical care or legal rights being affected. I understand that even if I withdraw from the STARFISH trial, data collected up to my time of withdrawal may still be used.	☐
3	I understand that relevant sections of my medical notes and data collected during the trial may be looked at by individuals from the STARFISH trial research team, representatives of The University of Birmingham, from regulatory authorities, or from the NHS Trust, where this is relevant to my taking part in this research.	☐
4	I have read and understood the information in the Participant Information Sheet and on the trial website about what happens to the personal data collected for this trial.	☐
5	I agree to my GP being informed of my participation in this trial.	☐
6	I understand and acknowledge that data collected that identifies me by name, on the consent form and the participant contact form, will be transferred from where it is collected and stored to the University of Birmingham.	☐
7	I understand that the information collected will be used for medical research and that I will not be identified in any way in the analysis and reporting of the results. I understand that even if I withdraw from the trial, information already collected about me may be included in the final analysis after being anonymised.	☐

8	I agree to take part in the STARFISH trial.	☐
9	**Optional:** I understand that the information collected about me may be used to support other related research in the future, and may be shared anonymously with other researchers.	☐
10	**Optional:** I agree to sharing my contact details with the STARFISH team, and to be contacted by them for up to 5 years after the trial, regarding my participation in the trial, further data collection, and to receive a summary of the trial results.	☐
11	**Optional:** I agree that information held in my medical notes, or maintained by NHS Digital and other central UK NHS bodies may be used to help contact me or provide information about my health status and medical history after the trial has ended. I agree to share my NHS number for this purpose.	☐
12	**Optional:** I agree to use the online hearing tests provided by a private company, HearX. I understand that I can opt out of this at any time during the study.	☐
13	**Optional:** I agree to share the results of my hearing tests, but not my name, with HearX for them to use to develop a new at-home hearing test. I understand that I can opt out of this at any time during the study.	☐

Name of participant	Signature	Date (e.g., 01/Jan/2021)
Name of person taking consent	Signature	Date (e.g., 01/Jan/2021)

When completed: Original to be kept in Investigator Site File, one copy for the participant, one copy for BCTU Trial Office, one copy to be filed in medical notes.

This study is funded by the National Institute for Health Research (NIHR) Heath Technology Assessment programme (project reference NIHR131528). The views expressed are those of the author(s) and not necessarily those of the NIHR or the Department of Health and Social Care.
