# Supplementary material for: Protocol for a multicentre randomised controlled trial of STeroid Administration Routes For Idiopathic Sudden sensorineural Hearing loss: The STARFISH trial
Source: PLoS One. 2024 Feb 29;19(2):e0290480. doi: 10.1371/journal.pone.0290480 (PMC10903811; doi:10.1371/journal.pone.0290480)
Supplement: S5 File — (PDF) [file pone.0290480.s006.pdf]

Oral Steroid Information Sheet

What are steroid tablets?
Steroid tablets are commonly used within the NHS to treat many different conditions, including sudden hearing loss.

You have been prescribed a steroid called Prednisolone that is taken in tablet form. You may need to take multiple tablets together every day; your pharmacist will advise you how to take the medication. The dose will be calculated for you and will be written on the box. Steroids are drugs that are similar to a hormone that the body naturally produces, and they act to reduce inflammation.

How should I take my medication?
Your prednisolone is best taken in the morning after breakfast, at around the same time each day. You should swallow tablets whole with water (do not chew them).

For maximum benefit, it is important that you take the tablets as directed every day, and complete the whole 7 day course of treatment.

Store tablets at room temperature, and out of sight and reach of children.

What if I miss a dose?
If you miss a dose it can be taken later in the day. If you miss a day, do not worry and do not try and catch up by taking the dose missed from the day before - just take the dose prescribed for that day.

What are the possible side effects?
Taking prednisolone can be associated with side effects which are mostly mild.
Less than half of people who take it have:

- Difficulty getting to sleep
- Mild changes to mood

Less than 1 in 10 people have:

- Temporary dizziness
- Indigestion

Very rare, less than 1 in 10,000 people have:

- Allergic reaction
- Severe change in mood

- Blood clots
- Bone fracture due to weakness or joint damage
- Spread of infection (sepsis)
- Bleeding from existing stomach ulcers

What if I become unwell?
If you experience these mild side effects such as altered sleep, changes to mood, dizziness and indigestion then, as long as you feel well, you can continue to take the prednisolone and complete the course. However, the course of prednisolone given in the STARFISH trial can be stopped early if you feel unwell or experience more severe side effects. For advice please contact the trial team at your local hospital or your GP in the event of problems.

If you experience the very rare side effect of an allergic reaction with a puffy, swollen face, tongue or body, which may cause shortness of breath or in very rare cases collapse**, stop taking the prednisolone Tablets and contact 111 or attend your local Emergency Department.**

What if I am diabetic?
You should discuss taking prednisolone with a member of the trial team, your GP or practice nurse. Prednisolone is likely to increase blood glucose level in patients with diabetes and it is important that this is monitored.

How can I monitor my hearing recovery?
If you would like to test your hearing at home, you can do this at the trial website: https://entintegrate.co.uk/hearing-test. After visiting the website you simply need to plug in headphones to a computer, tablet or smartphone and follow the instructions on screen. The website allows you to assess your own progress whenever you want, and it helps the trial team to build a picture of hearing recovery following steroid treatments.

Treatment record
The record below is purely to help you remember to take your steroids each day. We are not asking you to return this to us at your appointments.

	Day of the week	Morning (recommend)	Afternoon/Evening
Day 1	____________________	▢	▢
Day 2	____________________	▢	▢
Day 3	____________________	▢	▢
Day 4	____________________	▢	▢

Day 5	____________________	▢	▢
Day 6	____________________	▢	▢
Day 7	____________________	▢	▢
