# Supplementary material for: Protocol for a multicentre randomised controlled trial of STeroid Administration Routes For Idiopathic Sudden sensorineural Hearing loss: The STARFISH trial
Source: PLoS One. 2024 Feb 29;19(2):e0290480. doi: 10.1371/journal.pone.0290480 (PMC10903811; doi:10.1371/journal.pone.0290480)
Supplement: S6 File — (PDF) [file pone.0290480.s007.pdf]

Intratympanic Steroid Information Sheet

What is Intratympanic Steroid Injection?

Intratympanic steroid injection is a technique widely used in the NHS already to treat sudden hearing loss and other conditions.

Steroids are drugs that are similar to a hormone that the body naturally produces, and they act to reduce inflammation. Intratympanic injection is a technique to inject steroids through the ear drum. A local anaesthetic is used before the injection, which means that it is usually not painful and patients do not normally have any problems during injection. Steroids are injected through the ear drum and into the middle ear space (see below). From here, they can be taken up into the cochlea, which is the part of the inner ear which senses hearing.

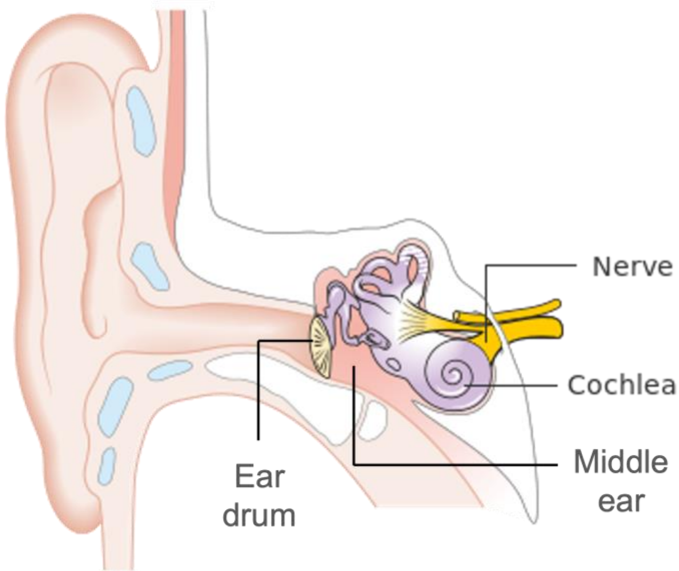

The outer, middle and inner ear (cochlea). The ear drum divides the outer from the inner ear.

How is the injection performed?

A local anaesthetic cream or spray is given to make your ear drum numb so that the injection is not usually painful - this takes between 5 and 30 minutes to work. Using a very thin needle and a microscope to guide it, the steroid is then injected through your ear drum and into your middle

ear. You will then remain lying down for 30 minutes after the injection so that the steroid is taken up into your inner ear.

What should I expect after my injection?

Most patients do not have any problems after injection, though a sensation of fullness in the ear is common and short-lasting. Some patients may experience a dull ear ache when the local anaesthetic wears off an hour later, and simple over-the-counter painkillers such as paracetamol or ibuprofen are effective if needed.

How do I look after my ear after my injection?

The injection leaves a small hole in your ear drum. In most people this heals up very quickly, within a few days. It is important that you are careful to keep your ear dry for at least a week following injection. When showering or bathing it is recommended that you block the ear canal with a piece of cotton wool covered by a thin film of Vaseline and placed in the opening of the ear canal.

How many injections will I have?

The standard treatment course is 3 injections spaced approximately 1 week apart. We will perform a hearing test before each injection. Should your hearing have recovered completely before the second or third injection, your doctor will discuss if further injections should be given.

What are the possible side effects?

Intratympanic steroid injections can be associated with side effects which are mostly mild.

Less than half of people:

- Mild ear discomfort

Less than 1 in 4 people:

- Temporary dizziness for a few minutes

Less than 1 in 20 people:

- Ear infection

Less than 1 in 100 people:

- Persistent hole in the ear drum

Very rare, less than 1 in 10,000 people:

- Allergic reaction
- Further hearing loss on the treated side

What if I become unwell?

Following intratympanic injection most patients experience either no or mild symptoms that resolve within an hour. However, if you experience an ear infection (painful ear with some discharge), further hearing loss or an allergic reaction (red painful ear) please contact the trial team at your local hospital or speak to your GP for advice.

How can I monitor my hearing recovery?

You will have hearing tests before each injection, and further tests at 6 and 12 weeks following your first treatment. You can ask the audiologist doing the tests to explain these to you.

If you would like to test your hearing at home, you can do this at the trial website: https://entintegrate.co.uk/hearing-test. After visiting the website you simply need to plug in headphones to a computer, tablet or smartphone and follow the instructions on screen. The website allows you to assess your own progress whenever you want, and it helps the trial team to build a picture of hearing recovery following steroid treatments.
