# Supplementary material for: Protocol for a multicentre randomised controlled trial of STeroid Administration Routes For Idiopathic Sudden sensorineural Hearing loss: The STARFISH trial
Source: PLoS One. 2024 Feb 29;19(2):e0290480. doi: 10.1371/journal.pone.0290480 (PMC10903811; doi:10.1371/journal.pone.0290480)
Supplement: S1 Protocol — (PDF) [file pone.0290480.s008.pdf]

Trial Protocol

STARFISH

A randomised controlled trial of STeroid Administration Routes For Idiopathic Sudden sensorineural Hearing loss

This protocol has regard for the HRA guidance and is compliant with the SPIRIT guidelines (2013)

**Version Number:**	3.0
**Version Date:**	10-Oct-2022

Protocol Development

Protocol Amendments				
The following amendments and/or administrative changes have been made to this protocol since the implementation of the first approved version.				
**Amendment number**	**Date of amendment**	**Protocol version number**	**Type of amendment**	**Summary of amendment**

Funding and Support in Kind	
**Funder**	**Financial and non-financial support given:**
National Institute for Health Research (NIHR)	£1,081,853.20
**Funding Scheme**	**Funder's reference number**
NIHR Health Technology Assessment Programme (HTA)	NIHR131528
**Funding call**	
19/128: Administration routes of steroids in the first-line treatment of idiopathic sensorineural hearing loss.	
This protocol was written in response to a commissioned call from the NIHR HTA programme (19/128). The funder of the trial will have no role in the trial design, data collection, data analysis or data interpretation.<br>This study is funded by the NIHR HTA programme as referenced above. The views expressed are those of the authors and not necessarily those of the NIHR or the Department of Health and Social Care.	

Protocol Sign Off

Chief Investigator Signature Page	
I, the Chief Investigator, confirm that I have read and agree with the following protocol, and that I will conduct the trial in compliance with the version of this protocol approved by the REC and any other responsible organisations.<br>I agree to ensure that the information contained in this document will not be used for any other purpose other than the evaluation or conduct of the clinical investigation without the prior written consent of the Sponsor.<br>I also confirm that I will make the findings of the trial publicly available through publication or other dissemination tools without any unnecessary delay and that an honest accurate and transparent account of the study will be given; and that any discrepancies from the study as stated in this and any subsequent approved protocol will be explained	
**Trial Name:**	STARFISH
**Protocol Version Number:**	Version: __ __
**Protocol Version Date:**	__ __ / __ __ __ / __ __ __ __
**CI Name:**	Dr James Russell Tysome
**Signature and date:**	____________________ __ __ / __ __ __ / __ __ __ __
**Sponsor statement:**<br>By signing the IRAS form for this trial, University of Birmingham, acting as sponsor, confirm approval of this protocol.	
**Compliance statement:**<br>This protocol describes the STARFISH trial only. This protocol should not be used as a guide for the treatment of participants not taking part in the STARFISH trial.<br>This trial will be conducted in compliance with the approved protocol, UK Policy Framework for Health and Social Care Research, Medicines for Human Use (Clinical Trials) Regulations 2004, Data Protection Act (2018) and the Principles of Good Clinical Practice (GCP) as set out in the UK Statutory Instrument (2004/1031) and subsequent amendments thereof.<br>Every care has been taken in the drafting of this protocol, but future amendments may be necessary, which will receive the required approvals prior to implementation.	

Principal Investigator (PI) Signature Page	
As Principal Investigator, I confirm that the following protocol has been agreed and accepted, and that I will conduct the trial in compliance with the approved protocol where this does not compromise participant safety.<br>I agree to ensure that the information contained in this document will not be used for any other purpose other than the evaluation or conduct of the clinical investigation without the prior written consent of the Sponsor.	
**Trial Name:**	STARFISH
**Protocol Version Number:**	Version: __ __
**Protocol Version Date:**	__ __ / __ __ __ / __ __ __ __
**PI Name:**	
**Name of Site:**	
**Signature and date:**	________________________ __ __ / __ __ __ / __ __ __ __

Administrative Information

Reference Numbers	
EudraCT number	2022-000085-17
Sponsor number	RG_21-145
ISRCTN reference number	<tbc>
IRAS reference number	1004878

Co-ordinating Centre Contact Details	
Birmingham Clinical Trials Unit<br>Public Health Building<br>University of Birmingham<br>Edgbaston<br>B15 2TT	STARFISH@trials.bham.ac.uk
Randomisation website/telephone number	https://www.starfish.bctu.bham.ac.uk<br>0800 9530274 *(available Monday to Friday, 09:00 to 17:00 UK time, except for bank holidays and UoB closed days)- for use in event of randomisation website being unavailable*
Trial website	www.birmingham.ac.uk/STARFISH
Trial social media	Twitter: @STARFISH_trial

Sponsor	
University of Birmingham	Dr Birgit Whitman, Head of Research Governance & Integrity
Research Support Group – Research Governance<br>Block C – Room 132, Aston Webb Building<br>Birmingham B15 2TT	0121 414 9144<br>researchgovernance@contacts.bham.ac.uk

Chief Investigator	
Dr James Russell Tysome	ENT Consultant
Ear, Nose and Throat<br>Cambridge University Hospitals NHS Foundation Trust<br>Hills Road<br>Cambridge<br>CB2 0QQ	01223 256051<br>jrt20@cam.ac.uk

Data Monitoring Committee	
Prof Mahmoud Bhutta (Chair)	ENT Surgeon, University of Sussex
Mr Manuel Loureiro	Audiologist, Brighton and Sussex University Hospitals
Dr Christopher Partlett	Medical Statistician, University of Nottingham

Trial Steering Committee	
Prof Rahul Kanegaonkar (Chair)	Professor of ENT, University of Christchurch Canterbury, independent
Dr Eldre Beukes	Audiologist, Anglia Ruskin University, Independent member
Prof Gary Barton	Professor in Health Economics, University of East Anglia, Independent member
Mr John Frangoudi	PPI, Independent member
Mrs Gulnaz Iqbal	Statistician, University of Warwick, Independent member

Mr Don McFerran	ENT Surgeon (retired), Colchester. President of the British Tinnitus Association, Sheffield, Independent member
Dr Matthew E Smith	Deputy Chief Investigator, non-independent
Dr James R Tysome	Chief Investigator, non-independent

**Trial Management Group**	
Dr James R Tysome	Chief Investigator, ENT Consultant, Cambridge University Hospitals NHS Foundation Trust
Dr Matthew E Smith	Deputy Chief Investigator (Co-chief investigator), Senior Clinical lecturer, University of Cambridge and Honorary ENT Consultant, Cambridge University Hospitals NHS Foundation Trust
Prof Manohar L Bance	Professor of Neurotology and Skull Base Surgery, Cambridge University Hospitals NHS Foundation Trust
Mrs Rachel Knappett	Senior Audiologist, Cambridge University Hospitals NHS Foundation Trust
Dr Debi Vickers	Principal Research Associate, The University of Cambridge
Dr Chris Schramm	General Practitioner, Primary Care Research Lead, Cambridge Community Service NHS Trust
Mr David White	Patient and Public Involvement Representative
Dr John Hardman	Chair of INTEGRATE, the UK ENT Trainee Research Network, ENT Registrar, Royal Marsden Hospital

Dr Chloe Swords	ENT trainee member of INTEGRATE
Prof Tracy Roberts	Professor of Health Economics and Head of Unit, University of Birmingham
Mr Samir Mehta	Senior Statistician Lead, University of Birmingham
Mr Yongzhong Sun	Trial Statistician, University of Birmingham
Dr Marie Chadburn	Trials Management Team Leader, University of Birmingham
Mrs Karen James	Trial Manager, University of Birmingham

ABBREVIATIONS

Abbreviation	Term
**AB**	Arthur Boothroyd word test
**AE**	Adverse Event
**AR**	Adverse Reaction
**BCTU**	Birmingham Clinical Trials Unit
**CI**	Chief Investigator
**CRFs**	Case Report Forms
**CTIMPs**	Clinical Trials of Investigational Medicinal Products
**dBHL**	Decibels (hearing loss)
**DCF**	Data Clarification Forms
**DMC**	Data Monitoring Committee
**DMP**	Date Management Plan
**DSA**	Data Sharing Agreement
**DSUR**	Development Update Safety Report
**ENT**	Ear, Nose and Throat
**GCP**	Good Clinical Practice
**GP**	General Practitioner
**HRA**	Health Research Authority
**HTA**	Health Technology Assessment
**HUI3**	Health Utility Index 3
**IB**	Investigator Brochure
**ICF**	Informed Consent Form
**ICECAP-A**	ICEpop CAPability measure for Adults
**IMP**	Investigational Medicinal Product
**ISF**	Investigator Site File
**ISSNHL**	Idiopathic sudden sensorineural hearing loss

**kHz**	Kilohertz
**MHRA**	Medicines and Healthcare Products Regulatory Agency
**MRS**	Maximum Recognition Score
**MP**	Monitoring Plan
**NHS**	National Health Service
**NICE**	National Institute for Health and Care Excellence
**NIHR**	National Institute of Health Research
**ONS**	Office for National Statistics
**PI**	Principal Investigator
**PIS**	Participant Information Sheet
**PPI**	Patient and Public Involvement
**PTA**	Pure Tone Average
**REC**	Research Ethics Committee
**RGT**	Research Governance Team
**RSI**	Reference Safety Information
**SAE**	Serious Adverse Event
**SAP**	Statistical Analysis Plan
**SAR**	Serious Adverse Reaction
**SmPC**	Summary of product characteristics
**SSNHL**	Sudden Sensorineural Hearing loss
**SSQ**	Speech, Spatial and Qualities of hearing questionnaire
**SUSAR**	Suspected Unexpected Serious Adverse Reaction
**TFI**	Tinnitus Functional Index
**TMF**	Trial Master File
**TSC**	Trial Steering Committee
**TMG**	Trial Management Group
**UK**	United Kingdom

**UAR**	Unexpected Adverse Reaction
**UoB**	University of Birmingham
**US**	United States
**USM**	Urgent Safety Measures
**VAS**	Visual analogue scale
**VRBQ**	Vestibular Rehabilitation Benefit Questionnaire

DEFINITIONS

Term	Description
Sudden sensorineural hearing loss (SSNHL)	Sensorineural hearing loss of >30dBHL over 3 contiguous pure-tone frequencies occurring within a 3-day period.

TRIAL SUMMARY

**Title**	STARFISH: A randomised controlled trial of STeroid Administration Routes For Idiopathic Sudden sensorineural Hearing loss
**Objectives**	**Primary Objectives:**<br>• To establish the relative effects of oral, intratympanic, or combined oral and intratympanic steroids on hearing recovery in idiopathic sudden sensorineural hearing loss (ISSNHL), when used as first line management.<br>**Secondary Objectives:**<br>• To complete a health economic assessment of the different routes of steroid administration.<br>• To use participant submitted data to explore the trajectory to hearing recovery.<br>**Economic Aims and Objectives**<br>To establish the cost-effectiveness of oral, intratympanic or combined oral and intratympanic steroids as the first line of treatment for ISSNHL.<br>**Exploratory Objectives**<br>To improve the early identification of ISSNHL in primary care.
**Trial Design**	A pragmatic, multicentre, assessor-blinded, parallel, three-arm intervention, superiority, randomised controlled trial (1:1:1) with an internal pilot.
**Participant Population/ Sample Size and Setting**	525 participants with sudden hearing loss will be recruited from ~75 NHS hospital Ear, Nose and Throat (ENT) units treating ISSNHL
**Eligibility Criteria**	**Inclusion Criteria**<br>• Adults aged 18 years or over<br>• Diagnosis of new-onset ISSNHL- sensorineural hearing loss of 30 decibels (dBHL) or greater occurring within a 3-day period and including 3 contiguous pure-tone frequencies (out of 0.5, 1.0, 2.0, 4.0 kilohertz (kHz)) confirmed with a pure tone audiogram.<br>• Onset of hearing loss within four weeks prior to randomisation<br>• English spoken as a first or second language<br>**Exclusion Criteria**<br>• Identified cause for hearing loss (not idiopathic) e.g. Meniere's<br>• Bilateral ISSNHL

	• Received prior steroid treatment for the same episode of ISSNHL<br>• Medical contraindication to high dose systemic steroids<br>• Previous history of psychosis<br>• On oral steroid therapy for another condition<br>• Known adrenocortical insufficiency other than exogenous corticosteroid therapy<br>• Hypersensitivity to the active substance or to any of the excipients<br>• Has a systemic infection unless specific anti-infective therapy is employed<br>• Has ocular herpes simplex<br>• Has ipsilateral acute or chronic active middle ear disease (including acute otitis media, chronic suppurative otitis media and cholesteatoma, excluding dry perforation)<br>• Does not have the capacity to provide written informed consent
**Interventions**	• Oral steroid (Prednisolone) 1mg/Kg/day up to 60mg/day for 7 days; Or<br>• Intratympanic steroid (Dexamethasone) three intratympanic injections 3.3mg/ml or 3.8mg/ml spaced 7±2 days apart; Or<br>• Combined oral (Prednisolone) and intratympanic (Dexamethasone) steroid as described above, with the first intratympanic injection occurring within four days of starting oral steroids.<br>All analysis will have the following two key group comparisons<br>o intratympanic steroid versus oral steroid<br>o combination oral and intratympanic steroids versus oral steroid
**Outcome Measures**	**Primary Outcome**<br>• The absolute improvement in pure tone audiogram average at 12-weeks following randomisation (calculated at 0.5, 1.0, 2.0 and 4.0 Kilohertz (kHz)<br>**Secondary Outcomes** (all at 6 and 12 weeks from randomisation unless stated)<br>• Functional hearing:<br>o Hearing related to speech: using The Speech, Spatial and Qualities of hearing scale (SSQ)<br>o Absolute improvement in hearing threshold at six weeks (calculated at 0.5, 1.0, 2.0, 4.0 kHz)<br>o Actual hearing thresholds at six and twelve weeks (calculated at 0.5, 1.0, 2.0, 4.0 kHz).<br>o High frequency hearing threshold across 4.0, 6.0 and 8.0 kHz<br>o Recovery of speech perception: using Arthur Boothroyd (AB) word lists scored by phoneme

	○ Extent of hearing recovery: using an established classification of recovery<br>● Associated symptoms: dizziness and tinnitus (Vestibular Rehabilitation Benefit Questionnaire & Tinnitus Functional Index)<br>● Adverse Events<br><br>**Optional**<br>● Weekly online home hearing tests (speech and pure tone thresholds)<br><br>**Health Economic Assessment**<br>● Health Utility Index 3<br>● ICEpop CAPability measure for Adults<br>● Resource usage

Trial Schema

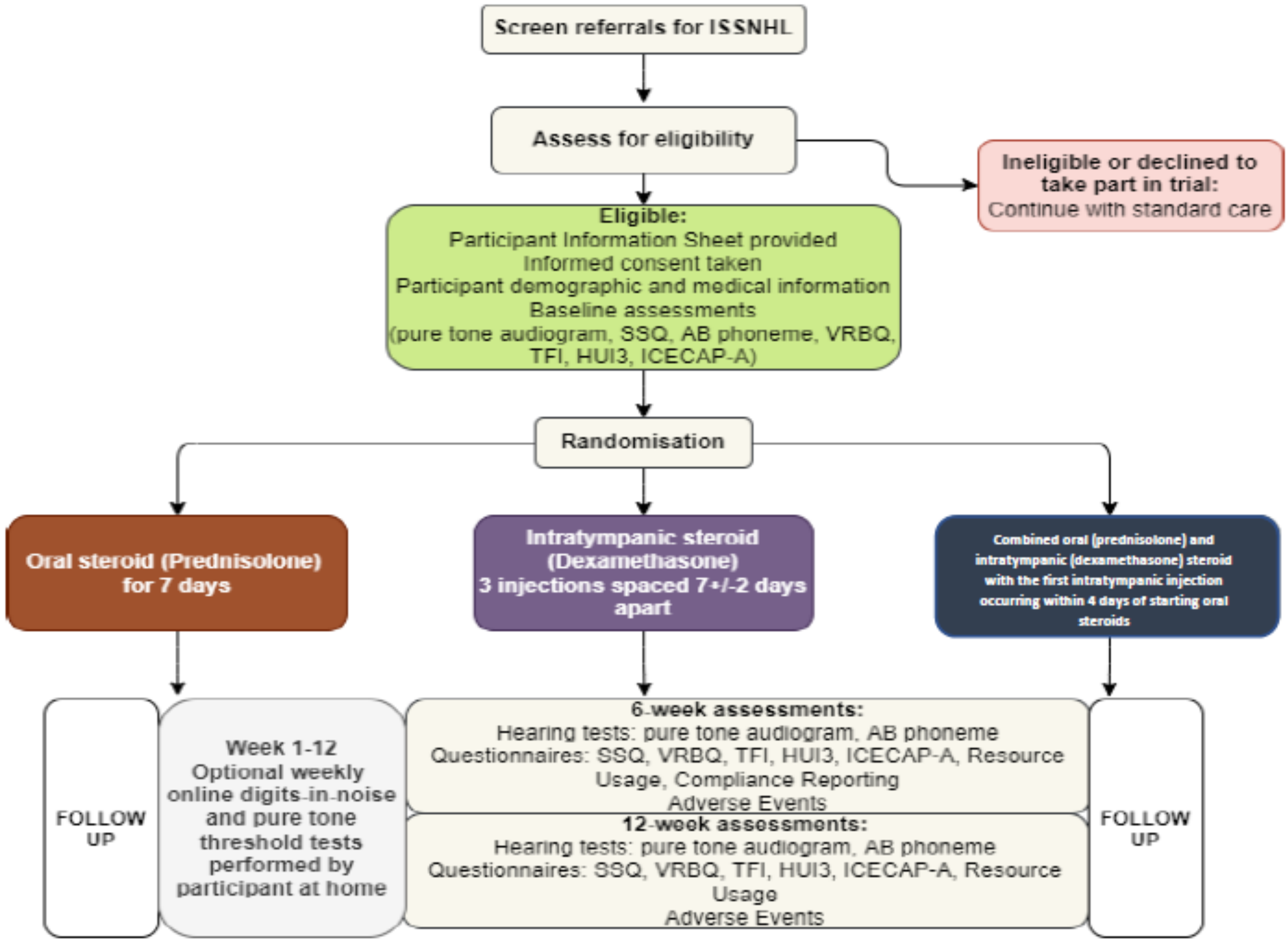

TABLE OF CONTENTS

BACKGROUND AND RATIONALE 20
- Background 20
- Trial Rationale 20
  - Justification for participant population 21
  - Justification for design 21
  - Choice of intervention 22
  - Justification for choice of primary outcome 22

AIMS AND OBJECTIVES 22
- Internal pilot objectives 22
- Main Trial Objectives 22
  - Clinical Aims and Objectives 23
  - Economic Aims and Objectives 23
  - Exploratory Objectives 23

TRIAL DESIGN AND SETTING 23
- Trial Design 23
- Trial Setting 23
- Assessment of Risk 23

ELIGIBILITY 23
- Inclusion Criteria 23
- Exclusion Criteria 24
- Co-enrolment 24

CONSENT 24
- Consent Procedure 24
- Consent documentation 25
- Ongoing consent 25
- Additional consent 25
- COVID-19 and trial process resilience 26

ENROLMENT, RANDOMISATION AND BLINDING 26
- Enhancing participant referral 26
- Identification 27
- Screening and enrolment 28
- Randomisation 28
  - Randomisation System 28
  - Randomisation Procedure 29
  - Randomisation Method 29
- Blinding 29

Informing the participant's GP 30

TRIAL INTERVENTION 30

Trial intervention and dosing schedule 30

Steroid dose calculation 30

Technique for intratympanic injection 30

Technical training and competency for the intervention 31

Pregnancy 32

Drug interaction or contraindications 32

Prohibited medications or treatments 32

Concomitant medications or treatments 32

Intervention modification or cessation 32

Continuation of intervention after the trial 33

Intervention supply and storage 33

Participant information 33

Adherence 33

Adherence criteria 33

Oral Steroid 34

Intratympanic Injection Steroid 34

Pure tone audiogram and speech testing 34

Accountability 34

OUTCOME MEASURES 34

Internal Pilot Outcome 34

Main Trial Outcomes 35

Primary Outcome 35

Secondary Outcomes 35

Outcome Procedures 38

Pure tone audiogram 38

AB word speech testing 38

Assessor training 39

Online hearing tests 39

TRIAL PROCEDURES 39

Loss to follow up definition 42

Participant withdrawal and changes in levels of participation 42

ADVERSE EVENT REPORTING 43

Definitions 43

Adverse Event recording – general 44

Adverse Event reporting in STARFISH 44

Serious Adverse Event reporting in STARFISH 45

Serious Adverse Events not requiring reporting to BCTU 45
Serious Adverse Events requiring non-expedited reporting to BCTU 46
Serious Adverse Events requiring expedited reporting to BCTU 46
SAE Reporting Process 46
Assessment of causality of an SAE 47
Assessment of expectedness of an SAE by the CI 48
Provision of follow-up information 49
Reporting SAEs to Third Parties 49
Data Monitoring Committee (DMC) 49
MHRA, REC and RGT 49
Urgent Safety Measures 50
Follow-up of Pregnancy Outcomes 50
DATA HANDLING AND RECORD KEEPING 50
Source Data 50
Case Report Form Completion 51
Participant completed Questionnaires 52
Home hearing test submission 53
Data Management 53
Data Security 54
Archiving 54
QUALITY CONTROL AND QUALITY ASSURANCE 55
Site Set-up and Initiation 55
Monitoring 55
Onsite Monitoring 55
Central Monitoring 55
Audit and Inspection 55
Notification of Serious Breaches 56
END OF TRIAL DEFINITION 56
STATISTICAL CONSIDERATIONS 56
Sample size 56
Analysis of outcomes 57
Primary outcome 57
Secondary outcomes 58
Planned subgroup analyses 59
Missing data and sensitivity analyses 59
Planned final analyses 59
HEALTH ECONOMICS 59

Within-trial economic evaluation 59
Data collection for the economic evaluation 60
Presentation of economic evaluation results 60

SUB-STUDIES 60

TRIAL ORGANISATIONAL STRUCTURE 61
Sponsor 61
Coordinating Centre 61
Trial Management Group 61
Trial Steering Committee 61
Data Monitoring Committee 61
Finance 61

ETHICAL CONSIDERATIONS 62

CONFIDENTIALITY AND DATA PROTECTION 62

FINANCIAL AND OTHER COMPETING INTERESTS 62

INSURANCE AND INDEMNITY 63

POST-TRIAL CARE 63

ACCESS TO THE FINAL TRIAL DATASET 63

PUBLICATION POLICY 63

REFERENCE LIST 65

APPENDICES 67

1 BACKGROUND AND RATIONALE

1.1 Background

Sudden sensorineural hearing loss (SSNHL) is the rapid onset of reduced hearing due to loss of function of the inner ear or hearing nerve. The cause is found in only 10-15% of participants, and can include infection, trauma and medication (1,2,3). In most cases, the cause is unknown or "idiopathic". Idiopathic sudden sensorineural hearing loss (ISSNHL) is usually unilateral, has an incidence of 5-20 per 100,000 and can result in permanent and complete hearing loss, although spontaneous recovery is seen in 32-65% of cases (2).

ISSNHL can have a profound impact on participants and their quality of life. Participants have described their experience of ISSNHL as 'terrifying' and 'isolating' and are motivated to try any treatment which may improve hearing recovery, even if it is invasive or time-consuming. Participants with ISSNHL usually see their General Practitioner (GP) fairly soon after symptom onset. A small proportion visit an Emergency Department or audiologist. If ISSNHL is suspected participants should be urgently referred to ENT specialists for assessment and treatment (3).

Evidence supports improved hearing recovery with early steroid treatment, via oral, intravenous or intratympanic routes (1, 2), with a combination of oral and intratympanic steroids possibly leading to superior hearing recovery (32). Identification of the most clinically and cost-effective route of administration of steroids as first-line treatment for ISSNHL is a high priority research recommendation from NICE (National Institute for Health and Care Excellence) guidelines [NG98] 'Hearing loss in adults: assessment and management'(3).

1.2 Trial Rationale

This study has been designed in response to a commissioned call by the National Institute for Health Research (NIHR) Health Technology Assessment (HTA) programme (see Appendix X for Commissioning Brief).

The best route of steroid delivery for hearing recovery in ISSNHL is unknown. Optimising treatment for ISSNHL is important for both participants and health resources as the condition results in reduced quality of life (4). Patient and Public Involvement (PPI) work by the trial team also identified a need for better diagnosis and access to specialist care if the management and hearing outcomes of individuals with ISSNHL are to be improved.

Members of our participant group have described that frequently, delay in diagnosis resulted in stress and anxiety that their hearing outcome could have been compromised. This is poorly represented in published studies on ISSNHL. Two main challenges in improving functional hearing recovery for participants with ISSNHL were identified:

1) Uncertainty around the most effective route of steroid administration
2) Delays in GPs identifying ISSNHL and arranging specialist care

While the first challenge is the main aim of the trial, we will also explore the second aim within the remit of this trial. The trial team have formed a GP advisory group, consisting of GPs and GP trainees (with and without clinical trials experience), with the aim of improving GP identification of ISSNHL and referral. Together with the Primary Care Academic Collaborative,

the team have designed action plans to maximise GP awareness, assessment and management of ISSNHL, which should aid referral and thus participant recruitment in the trial.

1.2.1 Justification for participant population

The target population in this trial is limited to adults with no upper age restriction. This is because ISSNHL is very rare in children, they have a different risk-profile to adults and there are implications around the practicalities of oral and intratympanic steroid use in children. .

The trial population includes women of child bearing potential. As with all drugs, corticosteroids should only be prescribed to pregnant women when the benefits to the mother and child outweigh the risks. Short-course high dose steroids are a low risk intervention in pregnant women. While old data had suggested that systemic corticosteroid use during pregnancy increases the risk of cleft lip/palate, preterm birth or low birth weight (5), recent studies have confirmed there is no independent association seen in the US National Birth Defect prevention Study (6,7). Maternal risks of corticosteroid specific side-effects such as hyperglycaemia and hypertension are similar to non-pregnant patients (e.g.) (8). Therefore, pregnant women are not excluded from this study. A discussion of the risks and benefits of steroids in pregnancy should take place if the study participant could be pregnant, as is standard clinical practice.

Participants with an identified cause for hearing loss or bilateral ISSNHL will be excluded as the intervention arms may not represent best care. Those with prior steroid treatment for the same episode of ISSNHL will also be excluded due to the risk of confounding treatment effects. Ipsilateral acute or chronic middle ear disease including acute otitis media, chronic suppurative otitis media and cholesteatoma will lead to exclusion as injected steroids may not reach the inner ear.

1.2.2 Justification for design

STARFISH is a three arm, superiority randomised, controlled trial. The three arms reflect the variation in current practice and the trial aims to elucidate which of these three arms is the optimal (superior) treatment. Randomised controlled trials are considered “gold standard” for evidence-based medicine.

Procedurally, the trial is designed to be in line with standard clinical practice as much as possible, to minimise burden on participants. The four week recruitment window from symptom onset reflects current practice, where more than half of UK surgeons would use intratympanic steroids up to one month after onset (9). Two meta-analyses suggest that the final hearing threshold may be independent of the delay in steroid treatment, for both primary (10) and secondary therapy (11), supporting the four-week treatment window, which is longer than some other trials adopt.

PPI suggested concern over recovery of hearing outweighs the anxiety associated with more invasive intratympanic steroid treatment, with randomisation acceptable to 80% of those surveyed. Reasons for the chosen minimisation variables include: there is a correlation between the hearing threshold before treatment and recovery (10); progressive spontaneous recovery may mean those treated later have already undergone some recovery; vertigo is a poor prognostic indicator (33).

Patient and public involvement in trial design led to the adoption of many of the outcome measures, including speech, tinnitus and dizziness questionnaires, and the optional participant-led home testing. The use of a modified classification system for recovery was suggested by participants, allowing the audiogram data to be translated into more relevant and meaningful measures to participants.

1.2.3 Choice of intervention

Oral steroid (Prednisolone) is included as an intervention as this is recommended in US guidelines (1), and used by most ENT clinicians across the UK (9,14). As the current standard of care, this represents control intervention.
Intratympanic steroid (dexamethasone) is included as a recent survey suggested 62% of UK ENT surgeons use intratympanic steroid injection in the treatment of ISSNHL (9) with dexamethasone the most commonly used steroid. 80% of surgeon's space injections 2-7 days apart (9), leading to the trial recommended schedule.

The third intervention is combined oral and intratympanic steroids, with the first intratympanic injection occurring within a period ±4 days of starting oral steroids.

Meta-analysis shows no evidence that one intratympanic steroid regime is superior (10), and so the most commonly used agent (dexamethasone) and frequency of injection (three) in the UK have been adopted. For the one third of ENT surgeons not currently using intratympanic injection for ISSNHL, the technique is easy to learn. The trial team will assist in training surgeons either remotely via videoconferencing and pre-recorded video materials, or on-site if required. This study will help disseminate new skills.

We have not incorporated a placebo arm as it would be unethical to withhold standard care with steroids (2,8), and during participant consultation, participants said they would not accept a placebo.

1.2.4 Justification for choice of primary outcome

The choice of primary end-point, the improvement of pure tone audiogram average, was recommended by an international consensus meeting (2) and is used in most studies in ISSNHL. Meta-analysis suggested that the time of endpoint measurement has no effect on the change in pure tone audiogram average (10). As hearing recovery is rarely seen after 12 weeks (15), the pure tone audiogram average at 12 weeks has been chosen as the primary endpoint.

2 AIMS AND OBJECTIVES

The aim is to determine whether oral, intratympanic, or combined oral and intratympanic steroids is the best first line treatment for ISSNHL.

2.1 Internal pilot objectives

The aim of the internal pilot is to assess the ability to open sites, recruit/randomise and retain participants. Section 8.1 details the criteria that will determine continuation of the trial beyond the pilot stage.

2.2 Main Trial Objectives

Primary Objectives:

- To establish the relative effects of oral, intratympanic, or combined oral and intratympanic steroids on hearing recovery in ISSNHL, when used as first line management.

Secondary Objectives:

- To complete a health economic assessment of different routes of steroid administration.
- To use participant submitted data to explore the trajectory to hearing recovery.

2.2.1 Clinical Aims and Objectives

To evaluate the clinical effectiveness of oral, intratympanic or combined oral and intratympanic steroids as the first line of treatment for ISSNHL.

2.2.2 Economic Aims and Objectives

To establish the cost-effectiveness of oral, intratympanic or combined oral and intratympanic steroids as the first line of treatment for ISSNHL.

2.2.3 Exploratory Objectives

To improve the early identification and onward referral of ISSNHL in primary care.

3 TRIAL DESIGN AND SETTING

3.1 Trial Design

A pragmatic, multicentre, assessor-blinded, parallel, three-arm intervention, superiority, randomised controlled trial (1:1:1) with an internal pilot.

3.2 Trial Setting

Approximately 75 UK NHS hospitals with ENT units treating ISSNHL. Site participation will be facilitated by working with the UK ENT trainee research network, INTEGRATE.

3.3 Assessment of Risk

All clinical trials can be considered to involve an element of risk and in accordance with Birmingham Clinical Trials Unit (BCTU) standard operating procedures, this trial has been risk assessed to clarify any risks relating uniquely to this trial beyond that associated with usual care.
A Risk Assessment has been conducted and concluded that this trial corresponds to the following categorisation:

Type A = No higher than the risk of standard medical care.

4 ELIGIBILITY

4.1 Inclusion Criteria

- Adults aged 18 years or over
- Diagnosis of new-onset ISSNHL- sensorineural hearing loss of 30 decibels (dBHL) or greater occurring within a 3-day period and including 3 contiguous pure-tone frequencies (out of 0.5, 1.0, 2.0, 4.0 kilohertz (kHz)) confirmed with a pure tone

audiogram occurring within a 3-day period (based on participant reported history)

- Onset of hearing loss within four weeks prior to randomisation
- English spoken as a first or second language

4.2 Exclusion Criteria

- Identified cause for hearing loss (not idiopathic)
- Bilateral ISSNHL
- Received prior steroid treatment for the same episode of ISSNHL
- Medical contraindication to high dose systemic steroids
- Previous history of psychosis
- On oral steroid therapy for another condition
- Known adrenocortical insufficiency other than exogenous corticosteroid therapy
- Hypersensitivity to the active substance or to any of the excipients
- Has a systemic infection unless specific anti-infective therapy is employed
- Has ocular herpes simplex
- Has ipsilateral acute or chronic active middle ear disease (including acute otitis media, chronic suppurative otitis media and cholesteatoma, excluding dry perforation)
- Does not have the capacity to provide written informed consent

4.3 Co-enrolment

Co-enrolment into other interventional trials will be considered by the Trial Management Group (TMG) on a case-by-case basis. The site should contact BCTU to seek advice prior to enrolling the participant.

5 CONSENT

It is the responsibility of the Principal Investigator (PI) or delegate to obtain written informed consent for each participant prior to performing any trial related procedure. This task can be delegated by the PI to other members of the local research team, if local practice allows and this responsibility has been documented in the STARFISH site signature and delegation log.

5.1 Consent Procedure

Participants with confirmed ISSNHL will be approached about the trial by their clinical care team during the standard clinical appointment following referral to secondary care.

A Participant Information Sheet (PIS) will be provided to facilitate this process, which may be presented electronically. Participants will also be invited to watch a short explanatory video on their own smartphone, tablet or hospital computer at https://entintegrate.co.uk/starfish. The PI or delegate will ensure that they adequately explain the aim of the trial, trial interventions, and the anticipated benefits and potential hazards of taking part in the trial to the participant. They will also explain that participation is voluntary and that the participant is free to decide to take part and may withdraw from the trial at any time without affecting their care.

The participant will be given sufficient time to read the PIS and discuss their participation with others outside of the clinical or research team. The participant will be given the opportunity to ask questions before electronically signing and dating the latest version of the Informed Consent Form (ICF). Paper copies of the PIS and ICF will also be available from the Trial Office and will be printed or photocopied onto the headed paper of the local NHS Trust.

Given the urgent need to commence treatment for the condition, randomisation and commencement of treatment should ideally occur on the same or next working day after written consent has been gained.

5.2 Consent documentation

If the participant expresses an interest in participating in the trial, they will be asked to electronically sign and date the latest version of the ICF, which will be made available to all participating sites online. The PI or delegate will then electronically sign and date the ICF via the trial system. A copy of the signed ICF will be given to the participant. Should participants wish to do so, they can receive a copy of the signed ICF electronically by consenting to provide an e-mail address, or alternatively a hard copy can be provided. A copy will be filed in the medical notes and a copy placed in the Investigator Site File (ISF). Once the participant is entered into the trial, the participant's trial number will be entered on the ICF maintained in the ISF. The participant's trial number will be linked against the consent form stored in the trial database. In addition, the participant understands and acknowledges that the signed ICF will be stored in the trial database at BCTU for review.

Details of the informed consent discussions will be recorded in the participant's medical notes. This will include date of discussion, the name of the trial, summary of discussion, version number of the PIS given to the participant, version number of ICF signed and date consent received. Where consent is obtained on the same day that the trial related assessments are due to start, a note should be made in the medical notes as to what time the consent was obtained and what time the procedures started.

Electronic copies of the PIS and ICF will be available from BCTU.

5.3 Ongoing consent

At each visit the participant's willingness to continue in the trial will be ascertained and documented in the medical notes. Throughout the trial, the participant will have the opportunity to ask questions about the trial.

Any new information that may be relevant to the participant's continued participation will be provided. Where new information becomes available which may affect the participants' decision to continue, participants will be given time to consider and if happy to continue will be re-consented. Re-consent will be documented in the medical notes. The participant's right to withdraw from the trial will remain.

5.4 Additional consent

The ICF contains optional additional statements for the participant to:

- Agree that information collected about them during the study may be used to support other related research in the future, and may be shared anonymously with other researchers.

- Agree to share their contact details with the STARFISH team, and to be contacted by them for up to 5 years after the trial, regarding their participation in the trial, further data collection, and to receive a summary of the trial results.
- Agree that information held and maintained by NHS Digital and other central UK NHS bodies may be used to help contact them or provide information about their health status and medical history after the trial has ended, and agree to share their NHS number for this purpose. This will allow investigators (subject to additional funding and ethical approval) to assess longer-term impact and health service usage data without needing further contact with the trial participants.
- Agree to use online hearing tests provided by a private company, HearX (full details are provided in section 8.3).
-
- Agree to share the results of their hearing tests, but not their name, with HearX for them to use to develop a new at-home hearing test.

5.5 COVID-19 and trial process resilience

Participant contact throughout the informed consent process and beyond, has been designed to coincide with routine care face-to-face assessments. Appointments will take place in person at the clinic, according to local and national guidance on COVID-19.

6 ENROLMENT, RANDOMISATION AND BLINDING

6.1 Enhancing participant referral

The trial will implement the following in order to try to enhance early referral of participants with ISSNHL:

GP awareness/education
A short educational article reviewing the diagnosis and management of ISSNHL will be submitted to GP targeted publications, designed to support GPs and practice nurses. This will be authored by clinical members of the TMG. It will be used as educational material to raise awareness of ISSNHL, and will not form part of the research.

Hospital site trainee leads will provide teaching at regional GP training events, covering the assessment of hearing loss, including ISSNHL. Slides will be provided by the trial team to facilitate this. The Primary Care Academic Collaborative will also contact their 350 UK-wide members regarding the trial, asking them to promote awareness of ISSNHL and the study in their practices.

ISSNHL diagnosis improvement - tuning fork assessments
We will disseminate a validated app developed by the STARFISH team to facilitate GP assessment of participants with suspected ISSNHL. This smartphone app and associated web-based app (entintegrate.co.uk) provides simple stepwise instructions on the technique for tuning fork tests, aids in interpretation, and then encourages users to refer based on the findings. If a tuning fork is not available during initial assessment or the participant is being reviewed remotely, an alternative to the Weber tuning fork test is the hum test, also described by the app. At each step, the app requests the GP to enter their findings using check boxes. These data are automatically entered into an algorithm, which on entry of the required information, informs the GP if the hearing loss may be sensorineural. The participant will not

have access to the app. No participant data is collected, thus avoiding data protection or NHS integration issues.

A short educational video will be made for professionals, including explaining the assessment of sudden hearing loss with tuning fork tests. This will be made available online on YouTube, accessed via a link in the app or via the website's search function. The video will also be hosted on the trial website, and cited in any GP targeted publications.

6.2 Identification

In line with NIHR guidance, the trial aims to improve care for underserved populations. For ISSNHL this includes those who do not present to healthcare promptly following onset of hearing loss, potentially reducing any benefit associated with steroid treatment.

Targeted online advertising

ISSNHL is common in younger adults who often search social media as a source of health information. Therefore, adverts have been developed to be displayed within Facebook explaining the importance of early assessment by a clinician after any sudden change in hearing, and highlight that the STARFISH trial may be relevant to them. The adverts will be displayed following an individual entering trigger search terms such as 'hearing loss' or 'deafness'.

Effectiveness of the advertising will be assessed after the trial has been open to recruitment for six months, and further advertising stopped if fewer than 5% of participants recruited to the study sought medical help as a result of the advert.

Advertising design

It is unclear what kind of messaging within the online adverts will best motivate participants to present to healthcare. Message 'framing' refers to the technique of presenting information in either a neutral (fact-based), a positive (gain-framed) or a negative (loss-framed) way. Gain-framed messages have been observed to be most effective when promoting prevention behaviours, e.g. smoking cessation and exercising regularly (16), whilst loss-framing is more effective for promoting detection behaviours, e.g. attending breast and colorectal cancer screenings (17).

As part of our aim to improve presentation to healthcare of those with ISSNHL, we have developed three different adverts that focus on using fact, gain or loss framed messaging. The three different adverts will be shown in rotation, with each shown for a month at a time, and will be used sequentially in rotation on Facebook. During the screening process, we will ask patients if they had seen the online advertising and whether it influenced them to seek medical advice with respect to their sudden hearing loss. The proportion of participants in the trial having responded to each of the three advertisements (based on timing of viewing) will be assessed to explore whether one form of message framing is superior at prompting participants to seek medical help. The results of this work will help to inform discussion at planned ISSNHL focus groups which will be set up as an independent study once the trial is underway. These focus groups will explore barriers and facilitators to accessing healthcare for ISSNHL.

Additionally to the advertising, the trial participant group and PPI lead manage a Facebook peer-support group for ISSNHL to provide more information on the condition and the trial.

Other advertising

Posters will be placed in community centres, primary care practices and high street audiologists, to explain to the public what ISSNHL is, and that urgent treatment may be needed. The process will be coordinated by local ENT trainees working with INTEGRATE. The poster will also be made available in electronic format for information screens within waiting rooms. This approach aims to reach those participants who may not normally present to health care and do not use social media.

The trial team will work with hearing related charities to advertise and raise awareness of the trial to their members, with the aim to cover as much of the target population as possible.

Potential eligible participants will be identified by clinicians at participating sites following referral to the ENT unit from GPs, Emergency Departments, and audiology services (hospital, community or high street). The participant's clinicians will then alert a member of the trial team at the site.

6.3 Screening and enrolment

An ENT clinician will confirm the diagnosis of ISSNHL via a pure tone audiogram. The potential participant will be asked by the clinical team if they are willing to speak to a member of the site research team to find out about the trial in more detail and to perform a complete eligibility assessment.

A medically qualified doctor who is delegated the task on the **STARFISH Site Signature and Delegation Log** will confirm eligibility. Investigators should consider the participant's medical history when determining if the sudden sensorineural loss is idiopathic. Pre-existing conditions such as ipsilateral Meniere's disease or multiple sclerosis that represent a high chance of being the aetiology for the hearing loss would make the participant ineligible for enrolment.

If the participants meet all the eligibility criteria and confirm that they are willing to take part in the trial, they will be asked to formally consent to participate in the trial as described in section 5.

Details of all participants approached about the trial will be recorded electronically on the **STARFISH Participant Screening/Enrolment Log**, which is part of the trial system. Since screening will occur prior to the participants providing consent, they will be identified by their sex, year of birth and date of screening only on the screening log.

6.4 Randomisation

6.4.1 Randomisation System

Randomisation will be provided by BCTU using a secure online system (available at https://www.starfish.bctu.bham.ac.uk), thereby ensuring allocation concealment. Unique login usernames and passwords will be provided to those who wish to use the online system and who have been delegated the role of randomising participants into the trial as detailed on the **STARFISH Site Signature and Delegation Log**. These unique login details must not be shared with other staff and in no circumstances should staff at sites access the system using

another person's login details. The online randomisation system will be available 24 hours a day, 7 days a week, apart from short periods of scheduled maintenance.

In the event of the online system being unavailable, a telephone toll-free randomisation service ((0044) 0800 953 0274) is available Monday to Friday, 09:00 to 17:00 UK time, except for bank holidays, government guided closures and University of Birmingham (UoB) closed days.

6.4.2 Randomisation Procedure

After participant eligibility has been confirmed and written informed consent has been received, the participant can be randomised into the trial using the online system. All questions and data items on the online randomisation form must be answered prior to a potential participant being randomised into the trial and trial number being issued.

Following randomisation, a confirmatory email will be sent to the local PI and the delegate who carried out the randomisation.

The local research team should add the participant to the **STARFISH Participant Recruitment and Identification Log**, which links participants with their trial number. The PI must maintain this document securely, which is not for submission to BCTU and it should be held in strict confidence.

6.4.3 Randomisation Method

Participants will be randomised at the level of the individual in a 1:1:1 ratio to either oral steroid only, intratympanic steroid only, or combined oral and intratympanic steroid treatment. A minimisation algorithm will be used within the online randomisation system to ensure balance in the treatment allocation over the following variables:

- Hearing loss severity pure tone average (PTA) at 0.5, 1, 2 and 4 kHz (mild/moderate less than 70dBHL; severe/profound 70dBHL or greater)
- Time since onset of hearing loss (≤14 days; >14 days)
- New vertigo since onset of hearing loss (participant reported yes; no)
- Site

To avoid the possibility of the intervention allocation becoming predictable, a 'random element' will be included in the minimisation algorithm. Full details of the randomisation specification will be stored in a confidential document at BCTU.

6.5 Blinding

Participant blinding is not required given the method of primary outcome assessment, which is objective from the perspective of the participant. PPI during study development also suggested that knowledge of treatment allocation is unlikely to (directly) influence the primary outcome. It is also not practical given the different routes of drug administration between the arms of the trial.

The audiologist assessing the primary outcome (the pure tone audiogram) will be blinded to the randomised treatment allocation (18) as the outcome can be considered subjective from the perspective of the assessor. The ENT team member reviewing the participant routinely at that time will examine the ear prior to audiological testing and confirm to the audiologist that

the ear is appropriate for testing, without disclosing the treatment allocation. Findings that may make the ear non-appropriate may be earwax or an infection. Wax will be removed to allow testing to proceed, and an infection will be treated and testing arranged at the nearest opportunity. If there needs to be a delay in testing, this will be recorded with the reason. The audiologist therefore will not need to look in the participant's ear.

In addition, where feasible, the pre-injection PTA tests should be conducted by an audiologist not recording later outcomes for the trial. The audiologist(s) performing the pre-injection audiogram and outcome testing will be recorded allowing assessment for outcome bias. In the small proportion where the same audiologist performs both tasks and would therefore be unblinded to the pre-treatment hearing thresholds.

6.6 Informing the participant’s GP

If the participant has agreed, the participant’s GP will be notified that they have enrolled in the STARFISH trial, using the **STARFISH GP Letter.**

7 TRIAL INTERVENTION

7.1 Trial intervention and dosing schedule

Participants will be randomised to receive:

- Oral steroid (Prednisolone as tablets) 1mg/kg/day up to 60mg/day for 7 days.

OR

- Intratympanic steroid (Dexamethasone) three intratympanic injections 3.3mg/ml or 3.8mg/ml spaced 7±2 days apart (see below for technique).

OR

- Combined oral (Prednisolone) and intratympanic (Dexamethasone) steroid as described above, with the first intratympanic injection occurring within four days of starting oral steroids.

7.2 Steroid dose calculation

The dose of prednisolone will be based on the participant’s body weight. Body weight should be measured in the clinic or estimated based on a weight recorded within 12 months used as a reference. In standard clinical practice, participants are not weighed prior to dosing, and a weight estimate used instead. This is appropriate given the safety profile of the intervention, and the lack of a requirement for strict dosing by weight. In many circumstances, participants will be seen out-of-hours when a requirement for weight measurement may delay urgent treatment. A dose of 1mg/kg/day should be calculated up to a maximum dose of 60mg/day. Dosing should be increased in increments of 5mg to allow for the use of 5mg tablets (e.g. a 50-54kg participant should receive 50mg). The recommended dose should be recorded in their medical records. It is recommended that oral prednisolone is taken in the morning.

7.3 Technique for intratympanic injection

Pre-injection testing: Immediately prior to the second and third intratympanic injections, a pure tone audiogram will be performed and analysed by the treating clinician.

Stopping the intervention early: If full recovery has been achieved, based on the pre-injection pure tone audiogram (return to within 10 dBHL of the unaffected ear at 0.5, 1.0, 2.0, 4.0kHz (if contralateral ear has no participant reported pre-existing loss)), the participant and clinician may jointly decide to omit further intratympanic injections. This will be recorded in the intervention Case Report Form (CRF) and the participant will continue within the trial.

Preparing the medication: Dexamethasone 3.3mg/ml or 3.8mg/ml should be administered depending on local availability and stored according to local policy. It is recommended that 1ml is drawn up in a 1ml syringe using a wide bore needle. A spinal needle (22-25 gauge) should then be attached to the syringe having removed the needle's central trochar. A slight bend at the hilt of the needle facilitates visualisation along the ear canal under a microscope.

Positioning the participant for injection: The procedure should be performed using a microscope or endoscope to visualise the tympanic membrane. The participant should lie recumbent on an examination table at an angle of <20 degrees, with the head turned slightly away from the side to be treated.

Topical anaesthesia of the eardrum: Any locally adopted method of local anaesthesia can be used, either in cream (most commonly EMLA (lidocaine 2.5% and prilocaine 2.5%)) or liquid form (most commonly co-phenylcaine spray (5% lidocaine + 0.5% phenylephrine) or xylocaine spray (10% lidocaine)). Local anaesthesia is not needed where there is a perforation of the eardrum.

- EMLA cream - The plunger is removed from a 2ml syringe to allow it to be filled with EMLA cream. The plunger is replaced and a blunt needle bent part way down the shaft to allow better visualisation during the application of EMLA cream to the eardrum under an outpatient microscope. It is important that the cream is in contact with the eardrum avoiding air gaps or bubbles. The anaesthetic can take 30 minutes to be effective.
- Co-phenylcaine or xylocaine spray – 4-6 sprays are administered directly into the ear canal with the study participant's head turned away to allow the liquid to remain in contact with the eardrum. The anaesthetic may take up to 10 minutes to be effective.

Intratympanic injection: Any local anaesthetic is removed using microsuction. Under visual guidance, the spinal needle should be passed through the tympanic membrane, ideally in the antero-superior quadrant in order to avoid the round window and ossicular chain. Dexamethasone is slowly injected, looking for the fluid level as it passes up the tympanic membrane. Once the middle ear is filled to the level of the needle, the needle can be withdrawn. Rapid filling of the middle ear should be avoided to reduce the risk of discomfort. The approximate volume of steroid injected into the middle ear should be recorded.

Positioning the participant after injection: Following the injection, the study participant should keep their head turned away from the injected ear and remain lying down in that position for 30 minutes to maximise uptake of steroid through the round window.

7.4 Technical training and competency for the intervention

Most clinicians will already practice intratympanic injection. However, some clinicians will not be familiar with the technique and all PIs and delegated staff performing intratympanic injection will be offered remote, or where necessary, face-to-face training.

Written material on the technique will be provided to sites. A video will also be available which will explain the participant positioning, setup and technical aspects of intratympanic injection. The training video will be available at: https://entintegrate.co.uk/starfish. Any clinician performing intratympanic injection within the trial will be required to review the training material.

Given that intratympanic injection is a simple procedure that is within the skill set of any ENT surgeon, only a self-assessment of competency and confirmation of training material review will be required. This will be recorded on a training log, which should be kept in the ISF and a copy forwarded to the STARFISH trial office. PIs will be responsible for approving trainee self-certification, and where ENT trainees have no or limited experience of the procedure, PI supervision is required.

7.5 Pregnancy

Investigators should follow standard clinical practice by asking if a female of child bearing potential is, or could be pregnant, before prescribing oral steroid treatment. This will allow the investigator to tailor the pre-intervention counselling to the individual, in line with routine clinical practice. .

7.6 Drug interaction or contraindications

7.6.1 Prohibited medications or treatments

Any medication which is contraindicated in people taking oral steroid (Prednisolone) or intratympanic injection steroid (Dexamethasone) according to the British National Formulary will be prohibited.

7.6.2 Concomitant medications or treatments

Oral and intratympanic steroid treatments have few drug interactions. In participants taking insulin or other medication for diabetes, additional care must be taken to regularly monitor blood glucose levels, and modify insulin dosing where indicated. Participants with diabetes will be provided with information on blood sugar control while on steroids or directed to their GP or diabetes specialist nurse.

7.7 Intervention modification or cessation

As specified in section 7.3, complete resolution of hearing may allow intratympanic steroid treatment to be terminated after only one or two treatments. A rare complication of intratympanic injection is further hearing loss. Where this is observed on pure tone audiometry, no further treatments will be given. In the rare event that a study participant chooses to change treatment type or has a significant side effect from oral steroids and is advised to modify or stop the treatment by their treating clinician this will be recorded. On rare occasions, changes to the intervention schedule may be required based on participant request or adverse events. The allocated interventions may be discontinued if a participant requests to cease treatment or cross over to another arm. Adverse events such as allergic reaction, or a change in patient status, such as development of poor blood glucose control may alter the risk-benefit profile for that individual and the treating clinician should discuss this with the patient and alter the provided intervention, if required. Details of treatment

adherence, treatment modifications or treatment discontinuation at the Investigator's discretion should be documented in the source data.

Any modification of the interventions, or cross over to another trial arm will also be recorded on the CRFs.

7.8 Continuation of intervention after the trial

Steroid treatment will be given in line with standard clinical practice during the trial. Neither oral nor intratympanic steroid courses are usually repeated, unless a new episode of SSNHL occurs. Once the participant completes or leaves the trial, they will not receive further steroid courses under the direction of the trial team, however they will be able to receive steroid treatment as part of their standard care.

While not part of the clinical trial, clinicians are encouraged to follow their usual local protocols for standard care of participants with ISSNHL in terms of the indication for MRI internal auditory meati to exclude a vestibular schwannoma and referral to audiology for discussion of hearing rehabilitation options.

7.9 Intervention supply and storage

Both oral steroid (Prednisolone) and intratympanic injection steroid (Dexamethasone) will be packaged, stored, prescribed and dispensed as per routine local practice. No trial specific supply, labelling or storage requirements will be needed.

7.10 Participant information

Participants will be provided with information leaflets specific to the intervention(s) they received, detailing precautions following intervention (e.g. keeping the ear dry to avoid infection), possible side effects, and where to seek further information or assistance if needed.

7.11 Adherence

7.11.1 Adherence criteria

A per protocol analysis will be performed for the primary outcome, and for the purposes of this, adherence to the oral steroid group will be defined as receiving prednisolone every day for 7 days, with treatment initiated within 4 weeks of symptom onset and not having the intratympanic treatment.

Adherence to intratympanic treatment group will be defined as receiving 3 intratympanic injections of dexamethasone (either 3.3 mg/ml or 3.8 mg/ml) spaced 7+/- 2 days apart, unless the course of injections was reduced to 1 or 2 injections on the basis of pure tone audiogram confirmed resolution of hearing loss or evidence of further sensorineural hearing loss, as well as not having received any oral steroid (prednisolone) with treatment initiated within 4 weeks of symptom onset. A reduction in course duration for intratympanic injection for any other reason will not be considered per protocol.

Adherence to the combined treatment group will require the criteria above to be met for both interventions. Additionally, if oral treatment is initiated within 4 weeks of symptom onset

then intratympanic treatment should be commenced up to four days preceding or following this.

7.11.2 Oral Steroid

Participants will be asked at their 6 week follow up visit whether they took the full course of treatment and this will be recorded on the relevant CRF.

7.11.3 Intratympanic Injection Steroid

Adherence will be documented in the relevant CRFs using information available in the medical notes.

7.11.4 Pure tone audiogram and speech testing

Adherence should be documented in the relevant CRFs using information available in the medical notes.

7.12 Accountability

Participants will receive their medication as per usual pharmacy practice at trial sites. As participants are receiving standard care, no additional monitoring is required.

8 OUTCOME MEASURES

8.1 Internal Pilot Outcome

The success of the 9-month internal pilot study will be based upon the recruitment rate, adherence to the treatment allocation and dropout rate. At 9 months, it is anticipated that the trial will have recruited 81 participants from at least 36 centres. For the trial to continue pre-defined stop-go criteria must be met. A traffic light system has been designed to determine continuation to the full trial:

Green: Recruitment rate ≥100%, adherence rate ≥90%, and dropout rate <5%. If all three criteria are met, the trial will continue with the protocol unchanged.
Amber: Recruitment rate 70-99%, adherence rate 70-89%, or dropout rate 5-10%. If one or more of the criteria are met, the trial will be reviewed to determine the changes (if any) that could be made to improve whichever criteria are not at the green level.
Red: Recruitment rate <70%, adherence rate <70%, or dropout rate >10%. If one or more of these criteria are met, this will be discussed with the Trial Steering Committee TSC) and funder regarding feasibility of the trial continuing.

Table 1: Internal pilot progression criteria

	Red	Amber	Green
**Recruitment threshold**	**<70%**	**70%-99%**	**≥100%**
**Adherence threshold**	**<70%**	**70%-89%**	**≥90%**
**Drop-out rate threshold**	**>10%**	**5-10%**	**<5%**

8.2 Main Trial Outcomes

8.2.1 Primary Outcome

- **The absolute improvement in pure tone audiogram average at 12 weeks** following randomisation (calculated at 0.5, 1.0, 2.0, 4.0 kHz). Conducted by an audiologist blinded to the treatment allocation.

8.2.2 Secondary Outcomes

All secondary outcomes will be measured at 6 and 12 weeks following randomisation, except where stated.

Functional hearing

- **Hearing related to speech: using The Speech, Spatial and Qualities of hearing scale (SSQ)**. SSQ is a validated measure, in the form of a short questionnaire, known to provide a good representation of the functional relationship with speech in everyday life and has been used to assess the disability of unilateral hearing loss seen in ISSNHL (19), providing disability scores associated with different aspects of hearing. **(Key secondary outcome)**
- **The absolute improvement in pure tone audiogram average at 6 weeks** following randomisation (calculated at 0.5, 1.0, 2.0, 4.0 kHz). Conducted by an audiologist blinded to the treatment allocation.
- **Actual hearing thresholds** measured by pure tone audiogram average following treatment initiation (calculated at 0.5, 1.0, 2.0, 4.0 kHz).
- **High frequency hearing threshold** measured by the absolute improvement in pure tone audiogram average across 4.0, 6.0 and 8.0 kHz.
- **Recovery of speech perception: using Arthur Boothroyd (AB) word lists scored by phoneme** (19). This speech testing will be carried out by an audiologist blinded to the treatment allocation. This measure is the most widely used in the UK and has a long history of routine use as an assessment tool for single sided hearing loss. Formal training will be provided to the audiologists. Calibration check and test setup will be provided at the training session. Continuing support can be provided throughout the trial where necessary.
- **Extent of hearing recovery: using an established classification of recovery** (complete/partial/none) based on pure tone audiogram and speech perception (1), shown in Table 2.

- **Time to hearing recovery: using online digits-in-noise and pure tone tests (*Optional and recommended weekly where done*).** Software has been developed for the trial by the HearX team, and will be iFramed on the trial website. Anonymised data entered by the participant is submitted from the client-side browser to HearX servers to store the tests. No data leaves the HearX servers except for API/csv download directly to BCTU. Weekly home testing of hearing will be encouraged in all groups. Regular testing will allow comparison of results with the pre-injection pure tone audiogram in the two intratympanic injection trial arms, and the 6 and 12 week assessments. The paired home and clinic test data will assist in validating the home tests, and provide added data on early hearing recovery in the oral steroid only group. Home testing is strongly supported by our ISSNHL PPI group and may provide valuable information for future trials of therapeutics.

Associated Symptoms

- **Dizziness: using the Vestibular Rehabilitation Benefit Questionnaire (VRBQ).** This is a 22 item participant completed tool and it provides a measure of the anxiety associated with dizziness, identified as an important aspect by the ISSNHL PPI group.
- **Tinnitus: using the Tinnitus Functional Index (TFI).** This is a 25 item participant completed questionnaire that is sensitive to changes in tinnitus severity and impact (20) and is recommended by NICE for this purpose (21).

Adverse Events (AEs)

- **Adverse events** relevant to the interventions will be recorded at 6 and 12 weeks (see section 10.3 for further details of timeline requirements of AE reporting).

Health Economic Assessment

- Two tools will be used to **assess health economics: the Health Utilities Index 3 (HUI3)**, a participant reported assessment of health-related quality of life suited to hearing loss (24,28), and **ICEpop CAPability measure for Adults (ICECAP-A)**, a participant reported measure of capability for the adult population (29).
- **Resource usage** over the past 12 weeks.

Table 2: The relationship between the pure tone audiogram and functional hearing assessments measured in the STARFISH trial, as well as American Academy of Otolaryngology – Head & Neck Surgery hearing recovery criteria (1).

Degree of hearing recovery	American Academy of Otolaryngology – Head & Neck Surgery criteria / Functional change	Outcome criteria
Unchanged	No recovery	● <10dB change in the pure tone audiogram
Partial Recovery Unaidable	Recovery to less-than-serviceable levels indicates an ear unlikely to benefit from traditional amplification.<br>Can use specialist hearing aid technology but unable to restore spatial hearing.	● ≥10dB improvement in pure tone audiogram<br>● AB phoneme maximum score <50%
Partial Recovery Aidable	Recovery to a serviceable level typically indicates that after recovery, the ear would be a candidate for traditional hearing amplification	● ≥10dB improvement in pure tone audiogram<br>● AB phoneme maximum score 50% or more
Full Recovery	Complete recovery requires return to within 10dBHL of the unaffected ear and	● Return to normal hearing (20dB or lower) or to within 10dB of the contralateral ear

Anticipated changes in other outcomes with unchanged hearing:

Unlikely to show a change in HUI3 score
Unlikely to show subjective changes in SSQ and other questionnaires

Anticipated changes in other outcomes partial recovery - unaidable:

Small but negligible improvements might be observed in the SSQ
Other questionnaires might indicate non speech-related improvements
Unlikely to show a change in Health Utilities Index 3 score

Anticipated changes in other outcomes partial recovery - aidable:

SSQ questionnaire will demonstrate functional improvement, which will be supported by other questionnaires
Measurable improvement in the Health Utilities Index 3 score

Anticipated changes in other outcomes with full recovery of hearing:

There will be a large change in the SSQ questionnaire and other questionnaires
Large improvements in the AB phoneme score
Large improvements in the Health Utilities Index 3 score

8.3 Outcome Procedures

8.3.1 Pure tone audiogram

Pure tone audiometry will be undertaken in clinics by audiologists blind to treatment allocation, in accordance with the British Society of Audiology Recommended Procedure for pure-tone air-conduction and bone conduction threshold audiometry with and without masking (2018) (25).

8.3.2 AB word speech testing

Audiologists blind to treatment allocation will perform speech testing, using AB wordlists on each ear individually. Supra-aural headphones (TDH-39) are routinely used, but insert earphones may be selected at the audiologist's discretion, taking care to select the appropriate routing and masking levels.

The ear with the best audiometric thresholds will be tested first to allow the participant to become familiarised with the task. The initial presentation level (Decibel Sensation Level, above PTA) will be around 30-40 dB above the PTA average across 250, 500 and 1000Hz. Masking of the contralateral ear will be applied in order to prevent the non-test ear being able to hear the speech material presented to the test ear. If the participant has a sensorineural hearing loss the masking noise will be presented 30 dB below the speech signal. The appropriate level of masking noise for sensorineural, conductive and mixed losses is derived from the following equation (26):
Ds + Em + max ABG nt -40
Where:

- Ds is the dial setting for presentation of speech to the test ear
- Em is effective masking calculated by measuring the difference in dial setting for speech presented to normal listeners at a level giving over 95% in quiet and the dial setting of noise presented to the same ear which leads to speech scores less than 10%. This factor has previously been measured and found to be 10 dB using a local biological calibration
- Max ABG nt is the maximum air-bone gap in the non-test ear 250 to 4000 Hz
- '40' comes from minimum interaural attenuation for masking in audiometry using headphones (note for inserts this would be 55 dB).

Each word is scored as a maximum of 3, one point for each correct phoneme. For the purpose of the study, the aim is to establish the Maximum Recognition Score (MRS), also known as PBmax for a phonemically balanced word list. Following completion of the first list, the presentation level should be increased in 10dB steps above the initial presentation level until one of the following:

- 3 points are obtained with scores over 95%
- Roll-over is clearly identified (a decrease in score at a minimum of two levels beyond the MRS)
- The maximum output of the audiometer is reached
- The participant reports discomfort

8.3.3 Assessor training

Training will be provided for audiologists not familiar with AB word testing. This will be recorded on a training log which should be kept in the ISF. This will take the form of written material and a short video on the testing technique. For any audiologists seeking additional help, remote one to one training will be provided via videoconferencing software. The AB word training material will be available at: https://entintegrate.co.uk/issnhl-rct

8.3.4 Online hearing tests

Bespoke software has been developed for the trial by the HearX Group to allow regular interval testing to chart the recovery of hearing. This will be hosted on the trial website, and will allow participants to self-test their hearing online at home. It will be recommended that participants complete the online tests weekly for the 12 week follow up period, however this part of the trial will be optional. Participants without internet access or the required skills, or those who prefer not to test at home will still be able to participate in the trial.

On visiting the website participants will be asked to enter their unique identifier provided at randomisation, and to connect headphones to the computer or device they are using. Headphones will be provided if participants do not have access to their own.

First participants will complete a digits in noise test that has been widely used in a World Health Organisation hearing screening app (34). The trial version will be modified to allow the collection of ear-specific data. Data from both ears will be collected, with the contralateral ear providing a measure of test-retest variability. Next participants will complete pure tone audiometry, again collecting ear-specific thresholds.

Participants will finish the online testing by marking the severity of their dizziness and the severity of their tinnitus on a 0-10 visual analogue scale (VAS) using a slider.

It will be recommended that participants use the same location and approximate time for the hearing tests, in an attempt to improve the consistency of results and to aid in regular testing. HearX has significant experience with digital remote hearing assessment, however additional validation procedures have been included in the study analysis plan to assess if online thresholds accurately reflect in-hospital hearing test results. Online hearing tests conducted within a few days of the pre-injection pure tone audiogram tests, or the 6 and 12 week pure tone and speech tests, will be compared to the audiologist performed hearing tests.

9 TRIAL PROCEDURES

Baseline

The following should be performed and/or collected:

- Informed consent
- Medical history
- Concomitant medication check
- Demographic information
- Pure tone audiogram (0.5-8.0kHz range) - should be performed within 3 days prior to commencement of treatment
- AB phoneme speech testing- recommended to be performed on same day as pure tone audiogram or if not practicable within next working day
- Participant completed questionnaires to include SSQ, VRBQ, TFI, HUI3, ICECAP-A
- Oral steroid arm and combination treatment arm- prescribe oral steroids

- Intratympanic injection arm and combination treatment arm - 1st intratympanic injection (for the combination arm, injection should be given within a period ±4 days of starting oral steroids).

Week 1 ±2 days after first injection
The following should be performed in the intratympanic injection arm and combination treatment arm:
- Pure tone audiogram (0.5-8.0kHz range)
- 2nd intratympanic injection

NOTE: complete resolution of hearing may allow intratympanic steroid treatment to be terminated after only one treatment

Week 2 ±2 days after first injection
The following should be performed in the intratympanic injection arm and combination treatment arm:
- Pure tone audiogram (0.5-8.0kHz range)
- 3rd intratympanic injection

NOTE: complete resolution of hearing may allow intratympanic steroid treatment to be terminated after only two treatments

Week 6 ±7 days after randomisation
The following should be performed and/or collected:
- Pure tone audiogram (0.5-8.0kHz range)
- AB phoneme speech testing
- Participant complete questionnaires to include SSQ, VRBQ, TFI, HUI3, ICECAP-A
- AE/SAE monitoring
- Compliance reporting
- Resource usage

Week 12 ±7 days after randomisation
The following should be performed and/or collected:
- Pure tone audiogram (0.5-8.0kHz range)
- AB phoneme speech testing
- Participant complete questionnaires to include SSQ, VRBQ, TFI, HUI3, ICECAP-A.
- Resource usage
- AE monitoring (intratympanic injection arm and combination treatment arm only for persistent perforation of the tympanic membrane)

For participants who have internet access and agree to use the online digits-in-noise and pure tone tests, they will be asked to perform the tests on the trial website every week, for 12 weeks from randomisation. In rare cases where the hearing tests are unable to be performed due to an infection in either ear, the infection should be treated as per standard local protocols and the audiogram performed as soon as the infection has cleared.

Table 3: Schedule of Assessments

	Visits						
			From first injection		From randomisation		
**Assessments**	**Screening**	**Baseline**	**2nd injection (week 1 ±2 days)**	**3rd injection (week 2 ±2 days)**	**Weekly**	**Week 6 ±7 days**	**Week 12 ±7 days**
Eligibility check	All						
Valid informed consent		All					
Relevant medical history taken		All					
Concomitant medication check		All					
Demographic information		All					
Randomisation		All					
Otoscopy		All	2,3	2,3		All	All
Pure tone audiogram (0.5-8.0kHz range)		All*	2,3	2,3		All	All
AB phoneme speech testing		All**				All	All
Online digits-in-noise test#					All#		
Online pure tone audiogram test#					All#		
Speech, Spatial and Qualities of hear scale (SSQ)		All				All	All
Vestibular Rehabilitation Benefit Questionnaire (VRBQ)		All				All	All
Tinnitus Functional Index (TFI)		All				All	All
Health Utilities Index 3 (HUI3)		All				All	All
ICECAP-A		All				All	All
Resource usage						All	All
Adverse Events monitoring						All	2,3&
Compliance reporting						All	
Oral steroid provision		1,3					
Intratympanic injection		2,3	2,3	2,3			

1 = for arm 1 (oral steroid), 2 = for arm 2 (intratympanic injection), 3 = for arm 3 (combined treatment)

#Optional, for participants who have internet access.

** should be performed within three days prior to commencement of treatment*

***recommended to be performed on same day as pure tone audiogram or if not practicable within next working day*

[&] Only persistent perforation of the tympanic membrane will be recorded at 12 weeks

9.1 Loss to follow up definition

Failure to collect primary outcome data at 12 weeks due to participant non-attendance at follow up.

9.2 Participant withdrawal and changes in levels of participation

Informed consent is defined as the process of learning the key facts about a clinical trial before deciding whether or not to participate. It is a continuous and dynamic process and participants should be asked about their ongoing willingness to continue participation at all visits. Participants should be aware from the beginning that they can freely withdraw (cease to participate) from the trial at any time. A participant may wish to cease to participate in a *particular* aspect of the trial.

Participants found to be ineligible post randomisation should be followed up according to all trial processes and will still have their data analysed unless they explicitly withdraw their participation.

The changes in levels of participation within the trial are categorised in the following ways:

- **No trial intervention:** the participant would no longer like to receive the trial intervention, but is willing to be followed up in accordance with the schedule of assessments and if applicable using any central UK NHS bodies for long-term outcomes (i.e. the participant has agreed that data can be collected and used in the trial analysis)
- **No trial related follow-up:** the participants no longer wish to attend trial visits in accordance with the schedule of assessments but is willing to be followed up at standard clinic visits and if applicable using any central UK NHS bodies for long-term outcomes (i.e. the participant has agreed that data can be collected at standard clinic visits and used in the trial analysis, including data collected as part of long-term outcomes)
- **No further data collection:** the participant is not willing to be followed up in any way for the purposes of the trial and does not wish for further data to be collected (i.e. only data collected prior to the withdrawal can be used in the trial analysis)

  The details of changes of levels in participation within trial (date, reason and category of status change) should be clearly documented in the source documents.

Participants can change their level of participation without giving a reason, although a reason would be very useful in the pilot to help assess whether it is related to the design of the trial.

BCTU should be informed of withdrawal or change in level of participation via the trial exit/change of status form.

10 ADVERSE EVENT REPORTING

10.1 Definitions

Table 4: Adverse event reporting definitions

**Severity Definitions**	Mild<br><br>Moderate<br><br>Severe	Awareness of signs or symptoms that do not interfere with the participant's usual activity or are transient and resolved without treatment and with no sequelae.<br><br>A sign or symptom, which interferes with the participant's usual activity.<br><br>Incapacity with inability to do work or perform usual activities.
**Adverse Event**	AE	Any untoward medical occurrence in a participant or clinical trial subject administered a medicinal product and which does not necessarily have a causal relationship with this treatment.<br>Comment:<br>*An AE can therefore be any unfavourable and unintended sign (including abnormal laboratory findings), symptom or disease temporally associated with the use of an investigational medicinal product, whether or not related to the investigational medicinal product.*
**Adverse Reaction**	AR	All untoward and unintended responses to an IMP related to any dose administered.<br>Comment:<br>*An AE judged by either the reporting Investigator or Sponsor as having causal relationship to the IMP qualifies as an AR. The expression reasonable causal relationship means to convey in general that there is evidence or argument to suggest a causal relationship.*
**Serious Adverse Event**	SAE	Any untoward medical occurrence or effect that:<br>● Results in death<br>● Is life-threatening*<br>● Requires hospitalisation or prolongation of existing hospitalisation<br>● Results in persistent or significant disability or incapacity<br>● Is a congenital anomaly/birth defect

		• Or is otherwise considered medically significant by the Investigator**
**Serious Adverse Reaction**	SAR	An Adverse Reaction which also meets the definition of an SAE
**Unexpected Adverse Reaction**	UAR	An AR, the nature or severity of which is not consistent with the applicable product information (e.g. Investigator Brochure (IB) for an unapproved IMP or (compendium of) Summary of Product Characteristics (SPC) for a licensed product).<br>When the outcome of an AR is not consistent with the applicable product information the AR should be considered unexpected.
**Suspected Unexpected Serious Adverse Reaction**	SUSAR	A SAR that is unexpected i.e. the nature, or severity of the event is not consistent with the applicable product information.<br>A SUSAR should meet the definition of an AR, UAR and SAR.

* The term life-threatening is defined as diseases or conditions where the likelihood of death is high unless the course of the disease is interrupted.
** Medical events that may not be immediately life-threatening or result in death or hospitalisation but may jeopardise the participant or may require intervention to prevent one of the other outcomes listed in the definitions above.

10.2 Adverse Event recording – general

The recording and reporting of AEs will be in accordance with the UK Policy Framework for Health and Social Care Research, the Principles of Good Clinical Practice (GCP) as set out in the UK Statutory Instrument (2004/1031; and subsequent amendments), the requirements of the Health Research Authority (HRA) and The Medicines for Human Use (Clinical Trials) Regulations 2004 and amendments thereof. Definitions for adverse event reporting are listed in Table 4 in Section 10.1.

It is routine practice to record AEs in the participant's medical notes and it is also recommended that this includes the documentation of the assessment of severity and seriousness and also of causality (relatedness) in relation to the intervention(s) in accordance with the protocol.

10.3 Adverse Event reporting in STARFISH

The safety profile for this trial population and interventions are well characterised so a strategy of targeted reporting of AEs will not affect the safety of participants.

Only the following AEs will be reported on the 6 week follow up CRF:

- Further sudden deterioration of hearing after intervention
- Affective disorders
- Psychotic reactions
- Stomach ulcer
- Acute ear infection (otitis externa or media)
- New dizziness onset or worsening after intervention
- New tinnitus onset or worsening after intervention
- Persistent ear drum perforation at 6 week review
- Allergic reaction to steroid preparation
- Allergic reaction to local anaesthetic

Only the following AEs will be reported on the 12 week follow up CRF:

- Persistent perforation of the tympanic membrane

The reporting period for AEs will be from commencement of trial treatment until the 12 week follow up visit (see above for further details of timeline requirements of AE reporting).

The reporting period for Serious Adverse Events (SAEs) will be from commencement of trial treatment until 6 weeks post treatment commencement. All AEs which meet the SAE definition should be reported as per section 10.4.

10.4 Serious Adverse Event reporting in STARFISH

All events which meet the definition of serious, must be recorded in the participant notes, including the causality and severity, throughout the participant's time on trial, including follow-up.

For all SAEs, the PI or delegate must do one of the following:

- **Record safety reporting-exempt SAEs** in the medical notes but **not report** them to BCTU on an SAE form as per Section 10.4.1
- **Report SAEs to BCTU in a non-expedited manner**. This can only be done for the pre-defined subset of AEs as per Section 10.4.2. These should be reported in line with the expected timeframes for other CRFs, i.e. within 4-weeks of becoming aware of the event.
- **Report SAEs to BCTU in an expedited manner** i.e. within 24 hours of the site research team becoming aware of the event. All SAEs not covered by the above two categories must be reported as per Section 10.5

Note: when an SAE occurs at the same hospital at which the participant is receiving trial intervention or is being followed up for trial purposes, processes must be in place to make the trial team at the hospital aware of any SAEs, regardless of which department first becomes aware of the event, in an expedited manner.

10.4.1 Serious Adverse Events not requiring reporting to BCTU

At whatever time they occur during an individual's participation, from consent to end of participant follow up, the following are not considered to be critical to evaluations of the safety of the trial:

a) Pre-planned hospitalisation
b) Hospitalisations lasting less than 24hrs

Such events are “safety reporting exempt”.

10.4.2 Serious Adverse Events requiring non-expedited reporting to BCTU

Where the safety profile is well established, the causal relationship between the intervention (or the participant’s underlying condition) and the SAE, may be known. That is, such events are protocol-defined as “expected”.

Such events should still be recorded in the participant’s medical notes and reported to BCTU on the SAE form (as per section 10.5) but it does not require expedited reporting (immediately on the site becoming aware of the event) since the assessment of expectedness for the specified events has been pre-defined. These should be reported in line with the expected timeframes for other CRFs, i.e. within 4-weeks of becoming aware of the event. These events are detailed in Table 5 below.

Table 5: Expected SAEs in STARFISH

Expected events related to oral steroids	Expected events related to intratympanic injection	Expected events related to both oral steroids and intratympanic injection
Blood glucose derangement requiring hospitalisation	Complete sensorineural hearing loss following injection	Allergic reaction requiring hospital treatment
Psychotic or affective disorder requiring hospitalisation		

10.4.3 Serious Adverse Events requiring expedited reporting to BCTU

All SAEs not listed in sections 10.4.1 and 10.4.2 must be reported to BCTU on a trial specific SAE form, within 24 hours of the site research team becoming aware of the event.

10.5 SAE Reporting Process

On becoming aware that a participant has experienced an SAE which requires reporting on an SAE form, the PI or delegate should report SAE to their own Trust in accordance with local practice and to BCTU.

To report an SAE to BCTU, the PI or delegate must complete, date and sign an SAE form via the STARFISH trial system using the information below in the timeline specified in sections 10.4.2 and 10.4.3. Any other relevant anonymised documents should be submitted to BCTU via the STARFISH trial mailbox (STARFISH@trials.bham.ac.uk).

The PI or delegate should also email STARFISH@trials.bham.ac.uk to make BCTU aware that an SAE has been submitted, along with any other relevant anonymised documentation.

Where an SAE Form has been completed by someone other than the PI (or medically qualified delegate) initially, the original SAE form will be required to be countersigned by the PI (or medically qualified delegate) to confirm agreement with the causality and severity assessments.

On submission of an SAE form, a unique reference number will be assigned. The site and BCTU should ensure that the SAE reference number is quoted on all correspondence. Site should also email the trial mailbox to inform BCTU that they have submitted an SAE. If the site has not received confirmation of receipt of the SAE within 1 working day of reporting, the site should contact BCTU.

Copies of the completed SAE form should be printed on resolution of the SAE and filed in the ISF.

10.5.1 Assessment of causality of an SAE

When completing the SAE form, the PI (or, throughout this section, a medically qualified delegate) will be asked to define the nature of the severity and causality (severity; see Table 4, relatedness; see Table 6) of the event.

In defining the causality, the PI must consider if any concomitant events or medications may have contributed to the event and, where this is so, these should be reported on the SAE form. It is not necessary to report concomitant events or medications which did not contribute to the event.

Table 6: Definition and category of causality for AEs

<table>
<tr><th>Category</th><th>Definition</th><th>Causality</th></tr>
<tr><td>Definitely</td><td>There is clear evidence to suggest a causal relationship, and other possible contributing factors can be ruled out</td><td rowspan="3">Related</td></tr>
<tr><td>Probably</td><td>There is evidence to suggest a causal relationship, and the influence of other factors is unlikely</td></tr>
<tr><td>Possibly</td><td>There is some evidence to suggest a causal relationship, however, the influence of other factors may have contributed to the event (e.g. the participant’s clinical condition, other concomitant events or medication)</td></tr>
<tr><td>Unlikely</td><td>There is little evidence to suggest there is a causal relationship; there is another reasonable explanation for the event (e.g. the participant’s clinical condition, other concomitant events or medication)</td><td rowspan="2">Unrelated</td></tr>
<tr><td>Not related</td><td>There is no evidence of any causal relationship</td></tr>
</table>

On receipt of an SAE form, BCTU will facilitate an independent review of causality of the SAE by the Chief Investigator (CI) or delegate. Where the CI is also the reporting PI an independent clinical causality review will be performed.

As per Table 6, all events considered to be ‘possibly’, ‘probably’, or ‘definitely’ related to the intervention will be reported by the trial office as ‘related’; all events considered at site to be ‘unlikely’ or ‘unrelated’ to the intervention will be reported by the trials office as ‘unrelated’. The same categorisation should be used when describing AEs and protocol-exempt SAEs in the source data.

An SAE judged by the PI or CI “or delegate(s)” to have a reasonable causal relationship (“Related” as per Table 6) with the intervention will be regarded as a related SAE (i.e., SAR).

10.5.2 Assessment of expectedness of an SAE by the CI

The CI or delegate will assess all SARs for expectedness with reference to the criteria in Table 7.

If the event is unexpected, it will be classified as a Suspected Unexpected Serious Adverse Reaction (SUSAR).

The CI will review all SAEs submitted and may request further information from the site research team for any given event to assist in this review.

Table 7: Definition of Expectedness

Category	Definition
**Expected**	An adverse event that is consistent with known information about the trial related procedures or that is clearly defined in the reference safety information:<br>Summary of product characteristics (SmPC) for oral prednisolone<br><br>SmPC for dexamethasone
**Unexpected**	An adverse event that is not consistent with known information about the trial related procedures or that is not clearly defined in the reference safety information.

10.5.3 Provision of follow-up information

Following reporting of an SAE for a participant, the participant should be followed up until resolution or stabilisation of the event. Follow-up information should be provided to BCTU via the trial database. The site should also email the trial mailbox to inform BCTU that follow up information has been submitted.

10.6 Reporting SAEs to Third Parties

10.6.1 Data Monitoring Committee (DMC)

The independent DMC may review any SAEs at their meetings.

10.6.2 MHRA, REC and RGT

BCTU will report details of all SARs (including SUSARs) to the Medicines and Healthcare Products Regulatory Agency (MHRA), Research Ethics Committee (REC) and UoB Research Governance Team (RGT) annually from the date of the Clinical Trial Authorisation, in the form of a Development Safety Update Report (DSUR).

In addition, BCTU will report a minimal data set of all individual events categorised as a fatal or life threatening SUSAR to the MHRA, REC and RGT within 7 days of being notified. Follow-up information will be provided within an additional 8 days.

All other events categorised as non-life threatening/non-fatal SUSARs will be reported within 15 days of being notified.

The MHRA, REC and RGT will be notified immediately if a significant safety issue is identified during the course of the trial.

Details of all SUSARs and any other safety issues which arise during the course of the trial will be reported to PIs. A copy of any such correspondence should be filed in the ISF and Trial Master File (TMF).

10.7 Urgent Safety Measures

The Clinical Trials Regulations make provision for the Sponsor and PIs to take appropriate Urgent Safety Measures to protect a research participant from an immediate hazard to their health and safety. This measure can be taken before seeking approval from MHRA and REC.

If any urgent safety measures are taken, BCTU shall immediately and in any event no later than 3 days from the date the measures are taken, give written notice to the MHRA and REC of the measures taken and the reason why they have been taken.

10.8 Follow-up of Pregnancy Outcomes

The trial population may include women of child bearing potential. Considering the level of risk and the factors outlined below, no additional monitoring of pregnancy or pregnancy outcomes is planned, other than would occur as part of routine clinical practice for the condition and treatments used.

- The trial treatments are well-established and will be used in accordance with their existing licences and routine clinical practice
- The treatment period is relatively brief
- Use of the trial treatments is not contraindicated in pregnancy
- The trial is unlikely to add to the known safety profile of the treatments under study in pregnancy
- Standard routes for reporting of suspected side effects (including the yellow card scheme) will be available to investigators, as in routine clinical practice

11 DATA HANDLING AND RECORD KEEPING

11.1 Source Data

Source data is defined as all information in original records and certified copies of original records of clinical findings, observations, or other activities in a clinical trial necessary for the reconstruction and evaluation of the trial. In order to allow for the accurate reconstruction of the trial and clinical management of the participants, source data will be accessible and maintained.

Some data variables may be entered directly onto the CRF, these are clearly identified and detailed in Table 8.

Table 8: Source Data

Data	Source
Audiometry Outcomes	The clinical notes provide the source data, transcribed at site onto the participant CRFs
Participant Reported Outcomes	The original participant-completed paper form is the source and will be forwarded directly to BCTU
Clinical event data	The original clinical annotation is the source document. This may be found on clinical correspondence, or electronic or paper participant records. Clinical events reported by the participant,

	either in or out of the clinic (e.g. phone calls), must be documented in the source documents.
Health economics data	Data will be completed directly on to the CRF via interview with the participant and this will constitute the source data.
Home hearing test data	Source data will be HearX server
Recruitment	The original record of the randomisation is the source. It is held on BCTU servers as part of the randomisation and data entry system.
Withdrawal	Where a participant expresses a wish to change their level of participation, the conversation must be recorded in the medical record.

11.2 Case Report Form Completion

CRFs should be completed for each individual participant.

The CRFs will include, but not be limited to, the forms listed in Table 9.

Table 9: List of trial specific CRFs

Form Name	Schedule for submission
Screening log	When a patient presents with ISSNHL and is considered for approach to participate in the STARFISH trial
Contact details form	Following consent
Randomisation CRF	At the point of randomisation
Clinic visit form	At baseline (i.e. following consent and randomisation);<br>At 2nd injection visit (if applicable);<br>At 3rd injection visit (if applicable);<br>At 6 weeks;<br>At 12 weeks.
Participant-complete booklet	At baseline (i.e. following consent and randomisation);<br>At 6 weeks;<br>At 12 weeks.

Serious Adverse Event Form	In accordance with section 10
Trial Exit/Change of status Form	At the point of becoming aware of withdrawal/change of status or death

Data should be submitted according to section 11.5 in a timely manner i.e. within four weeks of submission schedule. If data has not been provided within four weeks of the submission schedule detailed in the above table then a reminder email will be sent to sites. If data is consistently not provided in this timeframe BCTU will directly contact the site to ascertain the reason for the delay. This may also be escalated to the site's senior management and can trigger a monitoring visit.

In all cases, it remains the responsibility of the PI to ensure that the CRFs has been completed correctly and that the data are accurate. This will be evidenced by the signature of the PI or delegate(s). The Site Signature & Delegation Log will identify all those personnel with responsibilities for data collection. The delegated staff completing the CRF should ensure the accuracy, completeness and timeliness of the data reported. This will be evidenced by signing and dating the CRF.

Data reported on CRFs will be consistent with the source data and any discrepancies will be explained. All missing and ambiguous data will be queried via a Data Clarification Form (DCF). Staff delegated to complete CRFs will be trained to adhere to trial specific working instructions on CRF Completion.

The following guidance applies to data:

- Rounding conventions - rounding should be to the nearest whole number: If the number you are rounding is followed by 5, 6, 7, 8, or 9, round the number up. (e.g. 3.8 rounded to the nearest whole number is 4). If the number you are rounding is followed by 1, 2, 3 or 4, round the number down. (e.g. 3.4 rounded to the nearest whole number is 3).
- Trial-specific interpretation of data fields – where guidance is needed additional information will be supplied.
- Entry requirements for concomitant medications (generic or brand names) – generic names should be used where possible.
- Repeat tests – the data used to inform clinical decisions should always be supplied. If a test is repeated, it is either to confirm or clarify a previous reading. Confirmatory tests should use the original test values.
- Protocol and GCP non-compliances should be reported to BCTU on awareness.

11.3 Participant completed Questionnaires

A list of participant-completed forms can be found in Table 10.

Table 10: A list of participant completed questionnaires

Name of questionnaires
Speech, Spatial and Qualities of hear scale (SSQ)
Vestibular Rehabilitation Benefit Questionnaire (VRBQ)
Tinnitus Functional Index (TFI)
Health Utilities Index 3 (HUI3)
ICECAP-A

All participant questionnaires will be completed by participants in the clinic, overseen by site staff. Questionnaires should generally be completed by the participant alone, however physical assistance in completing the form can be given by the research staff or the participant's friends and relatives where appropriate. In such circumstances, questions are to be read to the participant verbatim and responses must not be led by the person assisting with the form completion. This requirement must be made clear when the participant's friends and relatives are providing the assistance. Participants should be encouraged to respond to all questions but can refuse to answer any, or all, of the questions should they wish. Where any questions are unanswered, research site staff should clarify with the participant that they have chosen not to respond specifically to the unanswered questions and that they have not simply missed them in error.

11.4 Home hearing test submission

Participants that consent to perform weekly hearing tests at home will access an online platform (HearX software) via the trial website in order to perform the hearing tests and submit their results. Participants will use their unique identification number to identify themselves. Data from the digits in noise test, pure tone thresholds and dizziness and tinnitus visual analogue scores will be collected, stored in the hearX server and transferred to BCTU by API or csv file.

11.5 Data Management

Processes will be employed to facilitate the accuracy and completeness of the data included in the final report. These processes will be detailed in the trial specific Data Management Plan (DMP) and include the processes of data entry and data queries.

Data entry will be completed by the site staff via a bespoke BCTU trial system with the exception of participant completed booklets which will be entered by BCTU staff. The data capture system will conduct automatic range checks for specific data values to ensure high levels of data quality. Queries will be raised using DCFs via the trial system, with the expectation that these queries will be completed by the site within 30 days of receipt. Overdue data entry and data queries will be requested on a monthly basis.

11.6 Data Security

The University of Birmingham has policies in place, which are designed to protect the security, accuracy, integrity and confidentiality of Personal Data.

The trial will be registered with the Data Protection Officer at UoB and will hold data in accordance with the Data Protection Act (2018 and subsequent amendments). BCTU has arrangements in place for the secure storage and processing of the trial data, which comply with UoB policies.

The Trial Database System incorporates the following security countermeasures:

- Physical security measures: restricted access to the building, supervised onsite repairs and storages of back-up tapes/disks are stored in a fireproof safe.
- Logical measures for access control and privilege management: including restricted accessibility, access controlled servers, separate controls of non-identifiable data.
- Network security measures: including site firewalls, antivirus software and separate secure network protected hosting.
- System Management: the System will be developed by the Programming Team at BCTU, and will be implemented and maintained by the Programming Team.
- System Design: the system will consist of a database and a data entry application with firewalls, restricted access, encryption and role based security controls.
- Operational Processes: the data will be processed and stored within BCTU. Anonymised data entered by the participant is submitted from the client-side browser to HearX servers to store the tests. No data leaves the HearX servers except for API/csv download directly to BCTU.
- System Audit: The System will benefit from the following internal/external audit arrangements:
    - Internal audit of the system
    - Periodic IT risk assessment
- Data Protection Registration: The UoB's Data Protection Registration number is Z6195856.

11.7 Archiving

All records created by following trial procedures and all documents listed in guidance relating to the conduct of the trial must be retained and archived for the specified period.

The TMF is normally composed of a sponsor file, held by the sponsor organisation, and an investigator site file, held by the site investigator. Documents are archived following regulatory requirements and local procedures.

Retained data should still be accurate, accessible and stored securely and confidentially.

It is the responsibility of the PI to ensure all essential trial documentation and source documents (e.g. signed ICFs, ISFs, participants' hospital notes, CRFs etc.) at their site are securely retained for the contractual period. Archiving will be authorised by BCTU on behalf of UoB following submission of the end of trial report. No documents should be destroyed without prior approval from the BCTU Director or their delegate.

The TMF will be stored at BCTU for at least 3 years after the end of the study. Long-term offsite data archiving facilities will be considered for storage after this time; data will be stored securely and confidentially for at least 25 years. BCTU has standard processes for both hard copy and computer database legacy archiving.

12 QUALITY CONTROL AND QUALITY ASSURANCE

12.1 Site Set-up and Initiation

All local PIs will be asked to sign the necessary agreements, including a **STARFISH Site Signature and Delegation log**, between the PI and BCTU and supply a current CV and GCP certificate. All members of the site research team are required to sign the Site Signature and Delegation Log, which details which tasks have been delegated to them by the PI. The Site Signature and Delegation Log should be kept up to date by the PI. It is the PI's responsibility to inform BCTU of any changes in the site research team.

Prior to commencing recruitment, each site will undergo a process of initiation, either a meeting or a tele/video conference, at which key members of the site research team are required to attend, covering aspects of the trial design, protocol procedures, adverse event reporting, collection and reporting of data and record keeping.

BCTU will provide each site with an ISF containing essential documentation, instructions, and other documentation required for the conduct of the trial. The PI or delegate is required to keep the ISF up to date throughout the trial.

12.2 Monitoring

The central and on-site monitoring requirements for this trial have been developed in conjunction with the trial specific risk assessment and are documented in the trial specific monitoring plan.

12.2.1 Onsite Monitoring

All sites will be monitored in accordance with the trial specific risk assessment and monitoring plan. Any monitoring activities will be reported to BCTU and any issues noted will be followed up to resolution. Additional on-site monitoring visits may be triggered. PIs and site research team will allow the BCTU staff access to source documents as requested. The monitoring will be conducted by BCTU/UoB staff.

12.2.2 Central Monitoring

BCTU will check received ICFs and CRFs for compliance with the protocol, data consistency, missing data and timing at a frequency and intensity as determined by the DMP. Sites will be sent DCFs requesting missing data or clarification of inconsistencies or discrepancies.

12.3 Audit and Inspection

The Investigator will permit trial-related monitoring, audits, ethical review, and regulatory inspection(s) at their site, providing direct access to source data/documents. The investigator will comply with these visits and any required follow up. Sites are also requested to notify BCTU of any relevant inspections or local audits.

12.4 Notification of Serious Breaches

In accordance with the Medicines for Human Use (Clinical Trials) Regulations 2004 and its amendments, the Sponsor of the trial is responsible for notifying the licensing authority in writing of any serious breach of the conditions and principles of GCP in connection with that trial or the protocol relating to that trial, within 7 days of becoming aware of that breach.

For the purposes of this regulation, a "serious breach" is a breach which is likely to affect;

- the safety or physical or mental integrity of the participants of the trial;
- the scientific value of the trial

Sites are therefore requested to notify BCTU of any suspected trial-related serious breach of GCP and/or the trial protocol as soon as they become aware of them. Where BCTU is investigating whether or not a serious breach has occurred, sites are also requested to cooperate with BCTU in providing sufficient information to report the breach to the MHRA where required and in undertaking any corrective and/or preventive action.

Sites may be suspended from further recruitment in the event of serious and persistent non-compliance with the protocol and/or GCP, and/or poor recruitment.

13 END OF TRIAL DEFINITION

The end of trial will be the date of the last data capture including resolution of DCFs. This will allow sufficient time for the completion of protocol procedures, data collection and data cleaning. BCTU will notify the Sponsor, MHRA and REC within 90 days of the end of the trial. Where the trial has terminated early, BCTU will inform the MHRA and REC within 15 days of the end of trial. BCTU will provide Sponsor, MHRA and REC with a summary of the clinical trial report within 12 months of the end of trial.

14 STATISTICAL CONSIDERATIONS

14.1 Sample size

Sample size has been calculated based on the primary outcome measure of absolute improvement in pure-tone audiogram. A typical gain in hearing threshold in the control group (oral steroid only) would be 25 dBHL, with standard deviation (SD) of 25 dBHL (2). As specified in published international criteria for ISSNHL (2), we have defined 10 dBHL as a minimum clinically important difference (MCID) for the sample size.
With 90% power and alpha of 0.025 (for the 3-arm trial design with two key group comparisons; see below), to observe a MCID of 10 dBHL with SD of 25 dBHL, the trial will require 157 participants per arm, or 471 participants in total. After allowing for a 10% attrition rate this gives a sample size of 175 per arm, or 525 in total for a 3-arm study.

<u>Key secondary outcome on spatial hearing</u>:
The secondary outcome of SSQ is validated to assess the disability associated with unilateral hearing loss seen in ISSNHL, and provides an additional measure of functional hearing for which the study is adequately powered. The mean SSQ total score for the control group (oral steroid only) is expected to approximate 4 points based on the average scores for an equivalent group in our own clinical sample (n=145 adults), with SD of 1.9 (19,27). A difference of 0.65 points for the SSQ total score has been defined as the MCID (19,27).

For the SSQ secondary outcome measure, 471 participants (525 recruitment target allowing for 10% attrition rate) will provide just under 80% power (alpha of 0.025, for the 3-arm trial design with two key group comparisons) to observe a MCID of 0.65 points with SD of 1.9 (effect size approximately 1/3 SD). The trial is therefore also adequately powered to demonstrate a difference in SSQ scores between intervention arms, complementing the primary outcome by indicating the difference in disability and functional impact of hearing recovery between interventions.

14.2 Analysis of outcomes

A separate Statistical Analysis Plan (SAP) will be produced and will provide a more comprehensive description of the planned statistical analyses. The reporting of the outcomes will be based on the guidelines as published in the Reporting of multi-arm parallel-group randomised trials (22). A brief outline of these analyses is given below.

<u>The objective of the trial is to test the superiority of three interventions</u>:

- Oral steroid
- Intratympanic steroid
- Combination oral and intratympanic steroid

<u>All analysis will have the following two key group comparisons</u>:

- Intratympanic steroid versus oral steroid
- Combination oral and intratympanic steroid versus oral steroid

Oral steroid group will be considered as the control group, i.e. reference category for all analyses. A comparison of intratympanic steroid versus combination oral and intratympanic steroid will also be performed as part of an exploratory analysis for all outcomes.

All primary analyses (primary and secondary outcomes including safety outcomes) will be by intention-to-treat (ITT), i.e. all participants will be analysed in the intervention group to which they were randomised irrespective of adherence to randomised intervention or other protocol deviation. As a secondary (or sensitivity) analysis, a per-protocol analysis will also be carried out for the primary outcome.

To maintain an overall 5% type I error rate, each comparison will be tested at a significance level of 2.5% to account for the increase in the risk of type I error associated with making two key comparisons. Therefore, for the primary outcome and all other secondary outcomes measures, a Bonferroni correction (23) will be applied to account for the increase in the risk of type I error associated with making two key comparisons (i.e. “intratympanic steroid versus oral steroid” and “combination oral and intratympanic steroid versus oral steroid”).

For all outcome measures, appropriate summary statistics and differences between groups, e.g. mean differences, relative risks, absolute differences will be presented, with two-sided 97.5% confidence intervals. Outcomes will be adjusted for the minimisation variables listed in section 6.4.3 and any baseline values (where applicable).

14.2.1 Primary outcome

The primary outcome is the absolute improvement in pure tone audiogram average at 12-week following randomisation (calculated at 0.5, 1.0, 2.0, 4.0 kHz). This data is collected at

baseline and at each follow-up visit. The data for this outcome is continuous and therefore will be summarised using the mean and standard deviation along with minimum and maximum values with respect to the intervention arms and overall at each time-point.
Difference between group means and associated 97.5% confidence intervals at each time-point will be estimated through the use of a repeated measures mixed-effects linear regression model. All assessment times (baseline and the post-treatment time points) will be included. Parameters allowing for participant, treatment group, time variable, the randomisation minimisation variables and the baseline score will be included (all as fixed effects). Time will be assumed to be a categorical (fixed) variable. To allow for a varying treatment effect over time, a time by treatment interaction parameter will be included in the model. Estimates of differences between groups at the 6 week and 12 week time-point will be taken from the model including this interaction parameter. Results will be presented as adjusted mean difference and 97.5% confidence interval. Longitudinal plots of the data over time will be constructed for visual presentation of the data.

14.2.2 Secondary outcomes

The secondary outcomes consist of continuous, time to event, binary, ordered categorical with more than two categories and count data types. For all secondary outcomes that are continuous in nature, they will be analysed using the same statistical method as described for primary outcome.

Binary outcomes: For those secondary outcomes that are binary in nature, data will be summarised as number and percentage of participants in each category by intervention arm. An adjusted relative risk and 97.5% confidence interval will be estimated from a log-binomial regression model and the p-value from the associated model will be produced and used to determine statistical significance. We will also present the adjusted risk difference and the corresponding 97.5% confidence interval.

Ordered categorical outcomes with more than two categories: For those secondary outcomes that are categorical in nature with more than two categories, data will be summarised as number and percentage of participants in each category by intervention arm. An adjusted odds ratio and 97.5% confidence interval will be estimated from an ordered logistic regression model and the p-value from the associated model will be produced and used to determine statistical significance.

Time to event outcome: For those secondary outcomes that are time to event data types, these will be compared between intervention arms using survival analysis methods. Kaplan-Meier survival curves will be constructed for visual presentation of time-to-event comparisons. A Cox proportional hazards model will be fitted to obtain an adjusted hazard ratio and 97.5% confidence interval. The p-value from the associated model will be produced and used to determine statistical significance.

Count data: For those secondary outcomes that are count data type, these will be analysed using a Poisson regression model (or negative binomial regression if there is evidence of overdispersion) with an offset for the length of time the participant was in the trial included in the model, to obtain an adjusted incidence rate ratio and 97.5% confidence interval. The p-value from the associated model will be produced and used to determine statistical significance.

14.2.3 Planned subgroup analyses

Subgroup analyses will be limited to the same variables used in the minimisation algorithm (see section 6.4.3) and performed on the primary outcome only. The effects of these subgroups will be examined by including an intervention group by subgroup interaction parameter in the regression model, which will be presented alongside the effect estimate and 97.5% confidence interval within subgroups. The results of subgroup analyses will be treated with caution and will be used for the purposes of hypothesis generation only.

14.2.4 Missing data and sensitivity analyses

Every attempt will be made to collect full follow-up data on all study participants; it is thus anticipated that missing data will be minimal. Participants with missing primary outcome data will not be included in the primary analysis in the first instance. This presents a risk of bias, and sensitivity analyses will be undertaken to assess the possible impact of the risk. In brief, this will include the use of multiple imputation using chained equations. Further sensitivity analysis will include a per-protocol analysis for the primary outcome only. Full details will be included in the SAP.

14.3 Planned final analyses

The primary analysis for the trial will occur once all participants have completed the 12 week assessment and corresponding outcome data has been entered onto the trial database and validated as being ready for analysis.

15 HEALTH ECONOMICS

A separate Health Economics Analysis Plan will be produced and will provide a more comprehensive description of the planned analyses. A brief outline of these analyses is given below.

15.1 Within-trial economic evaluation

A within-trial based economic evaluation will explore the cost-effectiveness of the 3 interventions being compared i) Oral steroid ii) Intratympanic steroid iii) Combination oral and intratympanic steroid.

A preliminary cost consequence analysis to establish whether any treatment shows dominance will be carried out. Dominance requires one strategy to be less costly with improved outcomes upon which the analysis is based. The economic evaluation will be based on three different outcomes: cost per case of any improvement in hearing; cost per case for the different increments of hearing improvement and; and cost per quality-adjusted life-year. Health-related quality of life will be assessed primarily using the HUI3, which is considered most appropriate in the field of hearing loss (24, 28). The HUI3 has eight dimensions (vision, hearing, speech, ambulation, dexterity, emotion, cognition and pain), and each dimension has five or six severity levels. These measures differ in terms of the description of health. We will also use ICECAP-A, which is a measure of capability for the general adult population for use in economic evaluation (29). The ICECAP-A focuses on wellbeing defined in a broader sense, rather than health. The measure covers attributes of wellbeing that were found to be important to adults in the UK. These utility data will be collected at baseline and 12 weeks. All other outcomes, improvements in hearing and increments of hearing improvement will be collected and assessed at 12 weeks.

15.2 Data collection for the economic evaluation

Resource use data will be collected prospectively from an NHS perspective, through CRFs in order to estimate the overall cost of the alternative interventions. Additional resource use such as that associated with additional appointments or interactions with the health service related to the intervention or adverse events will also be collected via a questionnaire to participants at 12 weeks. The primary analysis will adopt the perspective of the health service and personal social services.

The main resource categories to be monitored include:
1. Resource use associated with the procedures in all arms of the trial e.g. personnel present and length of time taken to carry out the procedure (Health Resource Group codes exist for the middle ear injections).
2. Resource use associated with adverse events and/or complications which may lead to further interactions with health services and/or additional medication beyond that expected (contacts with community and social care services e.g. GP).
3. We will also seek to record resources for procedure-specific training required for ENT surgeons and report this separately.

In order to value health care resource use to estimate the overall cost of each trial arm, unit costs will be applied to each resource item. Information on unit costs will be obtained from key UK national sources, such as the NHS reference costs, the Unit Costs of Health and Social Care, the British National Formulary, and the Office for National Statistics (ONS) (30).

15.3 Presentation of economic evaluation results

Initially, the base-case analysis for the within-trial analysis will be framed in terms of cost consequences, reporting data in a disaggregated manner on the incremental cost and the important consequences as assessed in the trial. The outcomes for the economic evaluation (Health Utilities Index 3 and ICECAP-A) will be presented in terms of Incremental Cost Effectiveness Ratios, and cost per quality-adjusted life-year, three months following treatment.

Other outcomes of cost per case of any improvement in hearing and cost per case for the different increments of hearing improvement will also be reported. Given the skewness inherent in most cost data and the concern of economic analyses with mean costs, we shall use a bootstrapping approach in order to calculate confidence intervals around the difference in mean costs (31).

The incremental economic analysis will be conducted on both the HUI3 and ICECAP-A, with confidence intervals generated to estimate uncertainty. The results of these economic analyses will be presented using cost-effectiveness acceptability curves to reflect sampling variation and uncertainties in the appropriate threshold cost-effectiveness value. We will also use both deterministic and stochastic cost effectiveness analyses to explore the robustness of these results to plausible variations in key assumptions and variations in the analytical methods used, and to consider the broader issue of the generalisability of the results.

16 SUB-STUDIES

There are no sub-studies in this trial.

17 TRIAL ORGANISATIONAL STRUCTURE

17.1 Sponsor

The Sponsor for this trial is University of Birmingham (UoB).

17.2 Coordinating Centre

The trial coordinating centre is Birmingham Clinical Trials Unit, based at UoB.

17.3 Trial Management Group

The TMG comprises individuals responsible for the day-to-day management of the trial: the CI, deputy CI, co-investigators, statisticians, trial team leader, trial manager, PPI members. The role of the group is to monitor all aspects of the conduct and progress of the trial, ensure that the protocol is adhered to and take appropriate action to safeguard participants and the quality of the trial itself. The TMG will meet sufficiently frequently to fulfil its function.

17.4 Trial Steering Committee

The TSC, comprising independent and non-independent members, will be established for the trial and will meet as required depending on the needs of the trial.

Membership and responsibilities are outlined in the TSC Charter. In summary, the role of the committee will provide oversight of the trial. The TSC will monitor trial progress and conduct, and provide advice on scientific credibility. The TSC will consider and act, as appropriate, upon the recommendations of the DMC. The TSC will operate in accordance with a trial specific TSC Charter.

17.5 Data Monitoring Committee

The role of the independent DMC is to monitor the trial data, and make recommendations to the TSC on whether there are any ethical or safety reasons as to why the trial should not continue or whether it needs to be modified. To this end, data on safety outcomes and (where appropriate) primary and major secondary outcomes will be supplied to the DMC during the trial. Reports will be supplied in confidence.

The DMC will operate in accordance with a trial specific charter, which will define the membership, roles and responsibilities of the DMC. The committee will meet at least annually as a minimum. Additional meetings may be called if needed e.g. recruitment is much faster than anticipated or a safety issue is identified.

17.6 Finance

The research costs of the trial are funded by the National Institute for Health Research (NIHR) Health Technology Assessment (HTA), reference NIHR 131528 awarded to James Tysome, Cambridge University Hospitals NHS Foundation Trust. The trial has been designed to minimise extra ‘service support’ costs for participating hospitals as far as possible. Additional costs, service support costs and excess intervention costs associated with the trial, e.g. gaining consent, are estimated in the Schedule of Events Cost Attribution Template (SoECAT). These costs should be met by accessing the Trust’s Support for Science budget via the Local Comprehensive Research Network.

18 ETHICAL CONSIDERATIONS

The trial will be conducted in accordance with the UK Policy Framework for Health and Social Care Research 2017, the applicable UK Acts of Parliament and Statutory Instruments (and relevant subsequent amendments), which include, but not limited to, the Medicines for Human Use Clinical Trials 2004 and Data Protection Act 2018.

This trial will be carried out under a Clinical Trial Authorisation in accordance with the Medicines for Human Use Clinical Trials regulations and Principles of GCP as set out in the UK Statutory Instrument (2004/1031; and subsequent amendments).

The protocol will be submitted to and approved by the REC prior to the start of the trial. All correspondence with the MHRA and/or REC will be retained in the TMF/ISF, and an annual progress report will be submitted to the REC within 30 days of the anniversary date on which the favourable opinion was given by the REC, and annually until the trial is declared ended. A trial specific risk assessment and monitoring plan will be developed before submission to the REC and will be reviewed regularly during the trial.

Before any participants are enrolled into the trial, the PI at each site is required to obtain the necessary local approval.

It is the responsibility of the PI to ensure that all subsequent amendments gain the necessary local approval. This does not affect the individual clinicians' responsibility to take immediate action if thought necessary to protect the health and interest of individual participants.

19 CONFIDENTIALITY AND DATA PROTECTION

Personal data and sensitive personal data recorded on all documents will be regarded as strictly confidential and will be handled and stored in accordance with the Data Protection Act 2018 (and subsequent amendments). Personal data categories that will be collected and analysed include name, date of birth, NHS number, email address, postal address and mobile number.

Participants will only be identified by their unique trial identification number and initials in any correspondence with BCTU. Participants will acknowledge the transfer and storage of their informed consent form to BCTU. This will be used to perform central monitoring of the consent process.

In the case of specific issues and/or queries from the regulatory authorities, it will be necessary to have access to the complete trial records. Representatives of the STARFISH trial team and sponsor may be required to have access to participants' medical notes for quality assurance purposes, but participants should be reassured that their confidentiality will be respected at all times. BCTU will maintain the confidentiality of all participant's data and will not disclose information by which participants may be identified to any third party.

20 FINANCIAL AND OTHER COMPETING INTERESTS

The interventions used in this study are already in standard use. There are no financial or other competing interests related to the results of this trial. Members of the TSC and DMC are required to provide declarations on potential competing interests as part of their membership of the committees. Authors are similarly required to provide declarations at the time of submission to publishers.

21 INSURANCE AND INDEMNITY

UoB has in place Clinical Trials indemnity coverage for this trial which provides cover to UoB for harm which comes about through the University's, or its staff's, negligence in relation to the design or management of the trial and may alternatively, and at UoB's discretion provide cover for non-negligent harm to participants.

With respect to the conduct of the trial at site and other clinical care of the participant, responsibility for the care of the participants remains with the NHS organisation responsible for the clinical site and is therefore indemnified through the NHS Litigation Authority.

UoB is independent of any pharmaceutical company, and as such it is not covered by the Association of the British Pharmaceutical Industry (ABPI) guidelines for participant compensation.

22 POST-TRIAL CARE

The interventions used in this trial are used in standard care. At the end of the trial, the participants will continue standard of care with their usual clinical care team.

23 ACCESS TO THE FINAL TRIAL DATASET

The final dataset will be available to members of the TMG and co-applicant group who need access to the data to undertake the final analyses.

Any request for data generated in this trial will be considered by BCTU. Data will typically be available 6 months after the primary publication unless it is not possible to share the data (for example: the trial results are to be used as part of a regulatory submission, the release of the data is subject to the approval of a third party who withholds their consent, or BCTU is not the controller of the data).

Only scientifically sound proposals from appropriately qualified Research Groups will be considered for data sharing. The request will be reviewed by BCTU Data Sharing Committee in discussion with the CI and deputy CI and, where appropriate (or in the absence of the CI and deputy CI) any of the following: the trial sponsor, TMG and independent TSC.

A formal Data Sharing Agreement (DSA) may be required between respective organisations once release of the data is approved and before data can be released. The data will be fully de-identified (anonymised) unless the DSA covers transfer of participant identifiable information. Any data transfer will use a secure and encrypted method.

24 PUBLICATION POLICY

The protocol will be made available online at https://entintegrate.co.uk/issnhl-rct, and within a trial registry.

On completion of the trial, the data will be analysed, and a Final Study Report prepared.

Results of this trial will be submitted for publication in a peer reviewed journal and the findings of the trial will be made public. The manuscript will be prepared by the writing group as defined in the trial publication plan. Any secondary publications and presentations prepared by Researchers must be reviewed and approved by the TMG. Manuscripts should be submitted to the TMG in a timely fashion and in advance of being submitted for publication, to allow time for review and resolution of any outstanding issues.

All PIs and Associate PIs submitting participant data will be included in authorship of the primary publication. INTEGRATE, the UK ENT Trainee Research Network will be included in the authorship list.

In all publications, authors should acknowledge that the trial was performed with the support of the NIHR HTA and UoB. Intellectual property rights will be addressed in the Clinical Trial Site Agreement between Sponsor and site.

Results will be made available to participants in a plain English summary and video.

25 REFERENCE LIST

1. Chandrasekhar SS, Tsai Do BS, Schwartz SR, Bontempo LJ, Faucett EA, Finestone SA, et al. Clinical Practice Guideline: Sudden Hearing Loss (Update). Otolaryngol Head Neck Surg. 2019;161(1_suppl):S1-S45.

2. Marx M, Younes E, Chandrasekhar SS, Ito J, Plontke S, O'Leary S, et al. International consensus (ICON) on treatment of sudden sensorineural hearing loss. Eur Ann Otorhinolaryngol Head Neck Dis. 2018;135(1S):S23-S8.

3. National Institute for Health and Care Excellence. NICE guideline [NG98] Hearing loss in adults: assessment and management. 2018.

4. Douglas SA, Yeung P, Daudia A, Gatehouse S, O'Donoghue GM. Spatial hearing disability after acoustic neuroma removal. The Laryngoscope. 2007;117(9):1648-51.

5. Carmichael SL, Shaw GM, Ma C, Werler MM, Rasmussen SA, Lammer EJ. Maternal corticosteroid use and orofacial clefts. Am J Obstet Gynecol 2007;197:585. e1-7; discussion 683–684, e1-7, https://doi.org/10.1016/j.ajog.2007.05.046.

6. Skuladottir H, Wilcox AJ, Ma C, Lammer EJ, Rasmussen SA, Werler MM, et al. Corticosteroid use and risk of orofacial clefts. Birt Defects Res A Clin Mol Teratol 2014;100:499–506. https://doi.org/10.1002/bdra.23248.

7. Bandoli G, Palmsten K, Forbess Smith CJ, Chambers CD. A review of systemic corticosteroid use in pregnancy and the risk of select pregnancy and birth outcomes. Rheum Dis Clin North Am 2017;43:489–502. https://doi.org/10.1016/j. rdc.2017.04.013.

8. Østensen M, Khamashta M, Lockshin M, Parke A, Brucato A, Carp H, et al. Anti-inflammatory and immunosuppressive drugs and reproduction. Arthritis Res Ther 2006;8:209. https://doi.org/10.1186/ar1957.

9. Lechner M, Sutton L, Ferguson M, Abbas Y, Sandhu J, Shaida A. Intratympanic Steroid Use for Sudden Sensorineural Hearing Loss: Current Otolaryngology Practice. The Annals of otology, rhinology, and laryngology. 2019;128(6):490-502.

10. Liebau A, Pogorzelski O, Salt AN, Plontke SK. Hearing Changes After Intratympanically Applied Steroids for Primary Therapy of Sudden Hearing Loss: A Metaanalysis Using Mathematical Simulations of Drug Delivery Protocols. Otol Neurotol. 2017;38(1):19-30.

11. Liebau A, Pogorzelski O, Salt AN, Plontke SK. Hearing Changes After Intratympanic Steroids for Secondary (Salvage) Therapy of Sudden Hearing Loss: A Meta-Analysis Using Mathematical Simulations of Drug Delivery Protocols. Otol Neurotol. 2018;39(7):803-15.

12. Mattox DE, Simmons FB. Natural history of sudden sensorineural hearing loss. The Annals of otology, rhinology, and laryngology. 1977;86(4 Pt 1):463-80.

13. Cvorovic L, Deric D, Probst R, Hegemann S. Prognostic model for predicting hearing recovery in idiopathic sudden sensorineural hearing loss. Otol Neurotol. 2008;29(4):464-9.

14. Shemirani NL, Schmidt M, Friedland DR. Sudden sensorineural hearing loss: an evaluation of treatment and management approaches by referring physicians. Otolaryngol Head Neck Surg. 2009;140(1):86-91.

15. Lee SS, Cho HH, Jang CH, Cho YB. Fate of sudden deafness occurring in the only hearing ear: outcomes and timing to consider cochlear implantation. J Korean Med Sci. 2010;25(2):283-6.

16. Bartels RD, Kelly KM, Rothman AJ. Moving beyond the function of the health behaviour: the effect of message frame on behavioural decision-making. Psychol Health. 2010 Sep;25(7):821-38.

17. Schneider TR, Salovey P, Apanovitch AM, Pizarro J, McCarthy D, Zullo J, Rothman AJ. The effects of message framing and ethnic targeting on mammography use among low-income women. Health Psychol. 2001 Jul;20(4):256-66.

18. British Society of Audiology. Recommended Procedure. Pure-tone air-conduction and bone-conduction threshold audiometry with and without masking. Reading: British Society of Audiology. 2011.

19. Gatehouse S, Noble W. The Speech, Spatial and Qualities of Hearing Scale (SSQ). International journal of audiology. 2004;43(2):85-99.

19. Boothroyd A. Developments in Speech Audiometry. British journal of audiology. 1968;2(1):3-10.

20. 30. Meikle MB, Henry JA, Griest SE, Stewart BJ, Abrams HB, McArdle R, et al. The tinnitus functional index: development of a new clinical measure for chronic, intrusive tinnitus. Ear and hearing. 2012;33(2):153-76.

21. National Institute for Health and Care Excellence. https://www.nice.org.uk/guidance/indevelopment/gid-ng10077. 2020.

22. Juszczak E, Altman DG, Hopewell S, Schulz K. Reporting of Multi-Arm Parallel-Group Randomized Trials: Extension of the CONSORT 2010 Statement. JAMA. 2019;321(16):1610-1620.

23. Vickerstaff, V., Omar, R. & Ambler, G. Methods to adjust for multiple comparisons in the analysis and sample size calculation of randomised controlled trials with multiple primary outcomes. BMC Med Res Methodol 19, 129 (2019).

24. Yang Y, Longworth L, Brazier J. An assessment of validity and responsiveness of generic measures of health-related quality of life in hearing impairment. Quality of life research : an international journal of quality of life aspects of treatment, care and rehabilitation. 2013;22(10):2813-28.

25.https://www.thebsa.org.uk/resources/pure-tone-air-bone-conduction-threshold-audiometry-without-masking/.

26. Coles RR, Priede VM. Masking of the non-test ear in speech audiometry. J Laryngol Otol. 1975 Mar;89(3):217-26.

27. Noble W, Gatehouse S. Interaural asymmetry of hearing loss, Speech, Spatial and Qualities of Hearing Scale (SSQ) disabilities, and handicap. International journal of audiology. 2004;43(2):100-14.

28. Grutters JP, Joore MA, van der Horst F, Verschuure H, Dreschler WA, Anteunis LJ. Choosing between measures: comparison of EQ-5D, HUI2 and HUI3 in persons with hearing complaints. Qual Life Res. 2007;16(8):1439-49.

29. Tsai TN, Huynh E, Peters TJ, Al-Janabi H, Clemens S, Moody A, et al. Scoring the Icecap-a capability instrument. Estimation of a UK general population tariff. Health Econ. 2015;24(3):258-69.

30. Personal Social Services Research Unit. https://www.pssru.ac.uk/pub/uc/uc2019/IntroductionUC2019.pdf.

31. Gray AM, Clarke PM, Wolstenholme JL, Wordsworth S. Applied Methods of Costeffectiveness Analysis in Healthcare. Oxford, UK: Oxford University Press; 2010.

32. Ahmadzai N, Kilty S, Cheng W, Esmaeilisaraji L, Wolfe D, Bonaparte JP, et al. A systematic review and network meta-analysis of existing pharmacologic therapies in patients with idiopathic sudden sensorineural hearing loss. PloS one. 2019;14(9):e0221713.

33. Rauch SD, Halpin CF, Antonelli PJ, Babu S, Carey JP, Gantz BJ, et al. Oral vs intratympanic corticosteroid therapy for idiopathic sudden sensorineural hearing loss: a randomized trial. JAMA. 2011;305(20):2071-9.

34. Potgieter JM, Swanepoel W, Smits C. Evaluating a smartphone digits-in-noise test as part of the audiometric test battery. S Afr J Commun Disord. 2018;65(1):e1-e6.

26 APPENDICES

Details of the commissioned call 19/128 from the NIHR HTA Programme: Administration routes of steroids in the first-line treatment of idiopathic sudden sensorineural hearing loss.

Research question:

What is the most effective route of administration of steroids as a first-line treatment for idiopathic sudden sensorineural hearing loss?

1. **Interventions:** oral steroids, intratympanic steroids or combined oral/intratympanic steroids as first line treatment. Applicants should specify and justify their choice of administration routes to be investigated, as well as their choice of steroid/s, and dosage.
2. **Participant group:** adults of any age presenting with new-onset idiopathic sudden sensorineural hearing loss. Applications are encouraged which include recruitment from geographic populations with high disease burden which have been historically underserved by research activity in this field.
3. **Setting:** ENT settings and any other suitable setting.
4. **Comparator:** the study should include a comparison between different routes of steroid administration. Double dummy techniques may be considered to mask the intervention.
5. **Study design:** applicants should consider the most appropriate randomised design to address the uncertainty between the treatment options. The study should include an internal pilot phase to test the acceptability of the proposed interventions, and the ability to recruit, randomise, and retain. Applicants are encouraged to refer to the international consensus statement on treatment of sudden sensorineural hearing loss and methodological guidance for future research (https://doi.org/10.1016/j.anori.2017.12.011).
6. **Important outcomes:** functional improvement and recovery. Applicants should explain and justify how these will be assessed.

   Other outcomes: changes in pure tone audiometry; adverse events; time to recovery; quality of life; acceptability; cost-effectiveness. Applicants may wish to consider the time between the onset of hearing loss and start of treatment and whether this would affect outcomes.

7. **Minimum duration of follow up:** 2 months.
   Longer term follow up: if appropriate, researchers should consider obtaining consent from participants to allow potential future follow up through efficient means (such as routine data) as part of a separately funded study.

Rational:

Sudden sensorineural hearing loss (SSNHL, also known as 'sudden deafness') is a rapid loss of hearing that can occur over a few hours or up to three days. It typically occurs in one ear only. The hearing loss in the affected ear can range from mid to total and can be temporary or permanent. In the vast majority of participants, the cause of SSNHL cannot be identified, and this condition is known as 'idiopathic sudden sensorineural hearing loss' (idiopathic SSNHL). SSNHL is considered an ENT emergency that requires immediate or urgent referral to specialist medical care, as stipulated in the NICE quality standard on hearing loss [QS185].

The mainstay of treatment currently consists of early initiation of steroids. These can include oral steroids (my mouth), intratympanic steroid injection (injection of steroids through the eardrum into the middle ear, also known as transtympanic injections), or a combination of both. Although UK and international guidelines recommend steroid treatments, there is currently a paucity of evidence assessing these treatments. Therefore, it is difficult to establish the most clinically and cost effective route of administration of steroids as first-line treatment for idiopathic SSNHL.

NICE, in the recent guideline on hearing loss [NH98], has therefore issued a high-priority research recommendation to answer this question. This suggested research is expected to inform future updates of key recommendations in this guideline.
