# Supplementary material for: Protocol for a multicentre randomised controlled trial of STeroid Administration Routes For Idiopathic Sudden sensorineural Hearing loss: The STARFISH trial
Source: PLoS One. 2024 Feb 29;19(2):e0290480. doi: 10.1371/journal.pone.0290480 (PMC10903811; doi:10.1371/journal.pone.0290480)
Supplement: Attachment — Submitted filename: Plos_STARFISH protocol_response 2.docx [file pone.0290480.s009.docx]

**PONE-D-23-24534: Response to peer review**

**Protocol for a multicentre randomised controlled trial of STeroid Administration Routes For Idiopathic Sudden sensorineural Hearing loss: the STARFISH trial**

We thank the academic editor and reviewers for their further review of our manuscript. We have addressed these individually below.

6. Review Comments to the Author

Reviewer #1: Thank you for the revision, I have no further issues to discuss. The authors have made all of the required changes and I would be happy to support publication.

Reviewer #2: Dear authors. Thank you for considering the reviewer comments. I fully understand that substantial modifications of the study protocol are not possible anymore. My question about my role as reviewer in this context was unfortunatly not answered by the editors. If focussing on the manuscript I have no further comments and wish good success when conducting the study!

Reviewer #3: In this study protocol, a three-arm randomized control trial with an initial pilot study is being proposed. The primary outcome will be absolute improvement in pure tone audiogram at 12-weeks. Cost effectiveness will also be compared among the three arms.

Minor revisions:
1- The standard statistical term for average is mean.

While we agree, pure tone average is the commonplace term in both research and clinical practice when describing the outcome of audiometry and so has been used in this work.

2- Indicate if adverse events will be collected according to a standardized format.

SAEs will be collected according to a standardised format on our SAE form and that AEs relevant to the interventions will be recorded according to a standardized format on our CRFs.

3- In the mixed-effect linear model indicate the type of underlying covariance structure that will be used or the method for selecting it.

We will be using a compound symmetry covariance structure for the mixed-effect linear model. This has been added to the revised manuscript.

4- For the adjusted Cox proportional hazards models, state the variables that will be used for adjusting or the method that will be applied to determine which variable will be included.

All analysis will be adjusted for the minimisation variables as fixed effects (except site which will be included as a random effect). This has been added to the revised manuscript.

5- Identify the software that will be used to capture the data as well as the software that will be used for the statistical analysis.

Statistical analysis will be undertaken in Stata version 18 (or higher) and/or SAS software, version 9.4 (or higher). This has been added to the revised manuscript.

6- To assist in the review process, add line numbers to the document.

Line numbers have been added
